# HIV-1 protease cleaves the serine-threonine kinases RIPK1 and RIPK2

**DOI:** 10.1186/s12977-015-0200-6

**Published:** 2015-08-22

**Authors:** Roland N. Wagner, John C. Reed, Sumit K. Chanda

**Affiliations:** Sanford Burnham Prebys Medical Discovery Institute, 10901 North Torrey Pines Road, La Jolla, CA USA; Department of Molecular Biology, University of Salzburg, Salzburg, Austria; Roche, Pharma Research and Early Development, Roche Innovation Center Basel, Basel, Switzerland

**Keywords:** HIV-1 protease, Receptor-interacting protein kinase, RIPK1, RIPK2, Proteolytic cleavage

## Abstract

**Background:**

HIV-1 protease (PR) is essential for viral infectivity as it cleaves Gag and Gag-Pol polyprotein precursors during viral maturation. Recent evidence suggests that cellular proteins can also be cleaved by PR, perhaps representing an important viral strategy to counter host defense mechanisms. Receptor-interacting protein kinase 1 (RIPK1) and RIPK2 belong to a family of serine/threonine kinases with conserved domain architecture and important functions in apoptosis, necrosis and innate immunity.

**Results:**

We found that RIPK1 and RIPK2 but not other members of the RIP kinase family are cleaved by HIV-1 PR. In RIPK1, we identified a putative PR cleavage site; a mutation at this site rendered RIPK1 resistant to PR cleavage. RIPK1 and RIPK2 were cleaved during HIV-1 infection of T cell lines or primary activated CD4^+^ T cells. Interfering with the viral life cycle at different stages by the addition of specific inhibitors against RT, integrase, or PR, completely prevented RIPK1 and RIPK2 cleavage. Cleavage of RIPK1 disrupted RIPK1/RIPK3 complex formation and RIPK1-mediated induction of NF-kB.

**Conclusions:**

These findings indicate that RIPK1 and RIPK2 are targets of HIV-1 PR activity during infection, and their inactivation may contribute to modulation of cell death and host defense pathways by HIV-1.

**Electronic supplementary material:**

The online version of this article (doi:10.1186/s12977-015-0200-6) contains supplementary material, which is available to authorized users.

## Background

HIV-1 has a compact genome that encodes only 15 proteins [1]. For successful replication, HIV must rewire the host’s cellular machinery to exploit numerous key host factors. Many aspects of the viral life-cycle, including entry, integration and release of viral particles, rely on a complex network of interactions between viral and host proteins. Cells targeted by HIV-1 express a number of proteins that function to restrict HIV-1 infection, including TRIM5α [2, 3], APOBEC3G [4–7] and Tetherin [8, 9]. Conversely, HIV-1 encodes a number of genes, such as Vif and Vpu, which counteracts these restriction factors.

The main function of the HIV-1 protease (PR) is to cleave the viral polypeptides Gag and Gag-Pol into mature proteins. Catalytic activity of PR is essential for the production of infectious progeny. PR has a complex specificity for substrate peptides with substrate shape rather than primary sequence being key for substrate recognition [10, 11]. PR is initially synthesized in an inactive form as part of the Gag-Pol precursor. During virus assembly and budding, which takes place on the cytoplasmic side of the plasma membrane, high concentrations of Gag and Gag-Pol lead to autoprocessing and the formation of a stable PR dimer with full catalytic activity [12–14].

Due to its degenerate substrate specificity, PR has also been found to cleave host cell proteins in vitro or during the course of infection (Additional file 1: Table S1). Cleavage of host factors by PR likely represents an important viral countermeasure against cellular restriction. The ever-growing list of host cell substrates includes cytoskeletal proteins [15], caspase-8 [16] and eukaryotic translation initiation factor 4G [17]. The list was further expanded by recent systematic analyses of interactions between PR and host cell proteins [18, 19].

RIPK1 and RIPK2 are members of the receptor interacting protein kinase (RIPK) family [20–23]. RIPKs are serine/threonine kinases that regulate a variety of cellular processes such as cell death and innate immune responses to viral and bacterial infection [24, 25]. All seven family members share a signature kinase domain (KD) but have different protein–protein interaction motifs. RIPK1 contains a C-terminal death domain (DD) that binds to death receptors such as tumor necrosis factor (TNF)-R1 and to DD-containing adaptor proteins. RIPK1 also contains an intermediate domain (ID) that features a RIP homotypic interaction motif (RHIM), which mediates interaction with RIPK3 [26] or other RHIM-containing proteins [27]. Activation of death receptors triggers the polyubiquitination of RIPK1 by inhibitors of apoptosis proteins (IAPs) [28, 29]. Ubiquitinated RIPK1 mediates activation of NF-κB and mitogen-activated protein kinases (MAPKs). In the absence of IAPs activity, de-ubiquitinated RIPK1 forms a complex with caspase-8 and promotes induction of apoptosis [30, 31]. Under circumstances where caspase-8 function is compromised, RIPK1 can recruit RIPK3 and initiate an alternative form of cell death called programmed necrosis, or necroptosis [24, 32, 33].

Along with its signature KD, RIPK2 also contains a caspase activation and recruitment domain (CARD). RIPK2 is a crucial adaptor protein downstream of the cytosolic pattern recognition receptors NOD1 and NOD2, which sense evolutionarily conserved bacterial peptidoglycan motifs [34, 35]. NOD2 also interacts with the mitochondrial outer membrane protein MAVS to stimulate interferon-inducing pathways involving IRF-family transcription factors [36]. Like TNFR1 complexes, NOD1 and NOD2 also interact with IAPs [37]. Activation of NOD1 and NOD2 leads to non-canonical ubiquitination and autophosphorylation of RIPK2, which in turn drives activation of NF-κB and MAPKs [38].

The RIPK family plays a key role in controlling viral infections; viral strategies that interfere with RIPK function have been described [39, 40]. Recently, an important role for RIPK1 in promoting activation of the NLRP3 inflammasome during RNA virus infection was demonstrated [41]. Furthermore, it was shown that RIPK1 kinase activity is crucial for induction of necroptosis in response to vaccinia virus infection [42]. RIPK2 function has also been linked to protection against influenza A virus infection [43]. However, at present no association between the RIPK family and retroviral infection have been established.

## Results

### HIV-1 PR binds RIP kinase family members

**Fig. 1:**
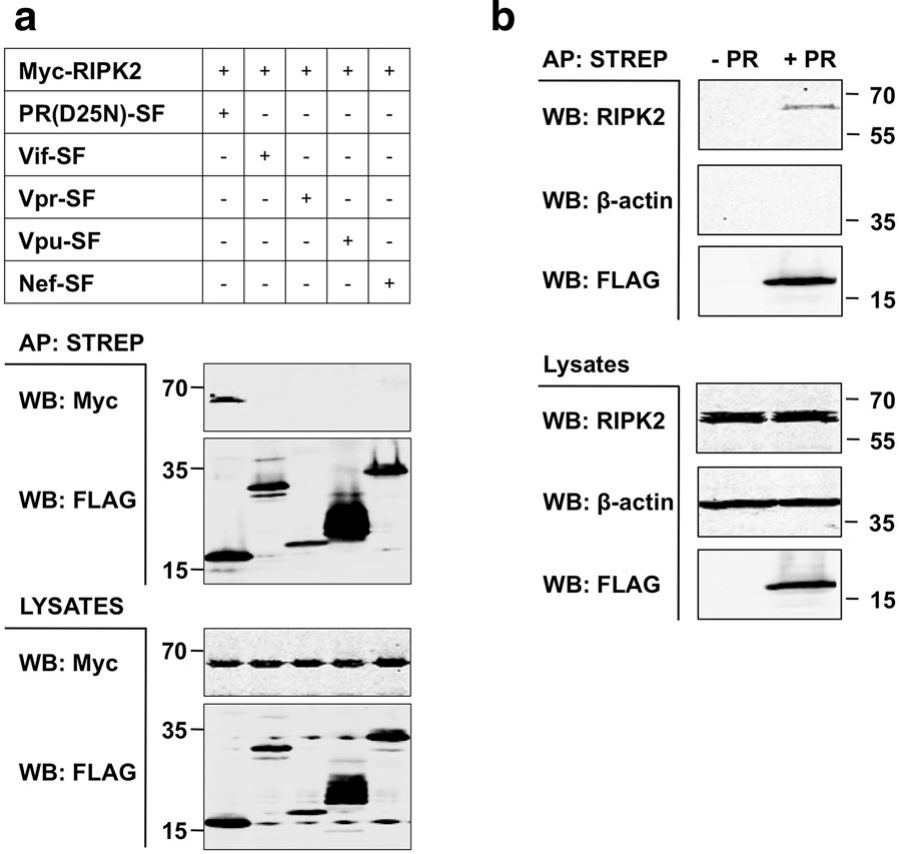
HIV PR binds RIP kinase family members. **a** HEK293T cells (1 × 10^6^) were transiently transfected with indicated expression plasmids (1 μg each). Various Strep-FLAG- (SF-) tagged HIV-1 proteins were expressed along with Myc-tagged RIPK2. After 24 h, proteins were affinity purified from cleared cell lysates with Dynabeads Streptavidin. Affinity purifications (APs) or total lysates were subjected to SDS-PAGE and Western blot (WB) analysis. Proteins were revealed using the indicated antibodies. **b** HIV PR binds to endogenous RIPK2. Catalytically inactive (D25N) SF-tagged HIV PR was expressed in HEK293T cells. Mock-transfected cells (-PR) were used as control. Complexes were purified as above. APs or total lysates were subjected to SDS-PAGE and Western blot (WB) analysis. Proteins were revealed using antibodies against RIPK2 (BD Transduction Laboratories) or β-actin (Sigma-Aldrich)

In their recent systematic analysis of HIV–human protein interactions, Jaeger et al. [19] found that HIV-1 PR bound to a number of human proteins including RIPK2. To independently confirm the reported RIPK2-PR interaction, we over-expressed and affinity purified (AP) epitope tagged proteins in HEK293T cells. Since over-expression of wild-type PR is cytotoxic [44–46], a catalytically inactive PR mutant (D25 N) was used in our studies. We observed binding of PR (D25 N) to RIPK2. This interaction was specific for HIV-1 PR as RIPK2 did not bind other HIV proteins, including Vif, Vpr, Vpu, or Nef (Fig. 1a). We also found that over-expressed PR (D25N) bound to endogenous RIPK2 but not to β–actin (Fig. 1b). Again, this result was specific for HIV-1 PR as none of the other HIV proteins (n = 12) bound to endogenous RIPK2 (Additional file 2: Figure S1). We found that PR can also interact with RIPK1, but not RIPK3 (data not shown). Taken together, these results show that PR can selectively recognize members of the RIP kinase family.

### RIPK1 and RIPK2 are cleaved by HIV-1 PR

**Fig. 2:**
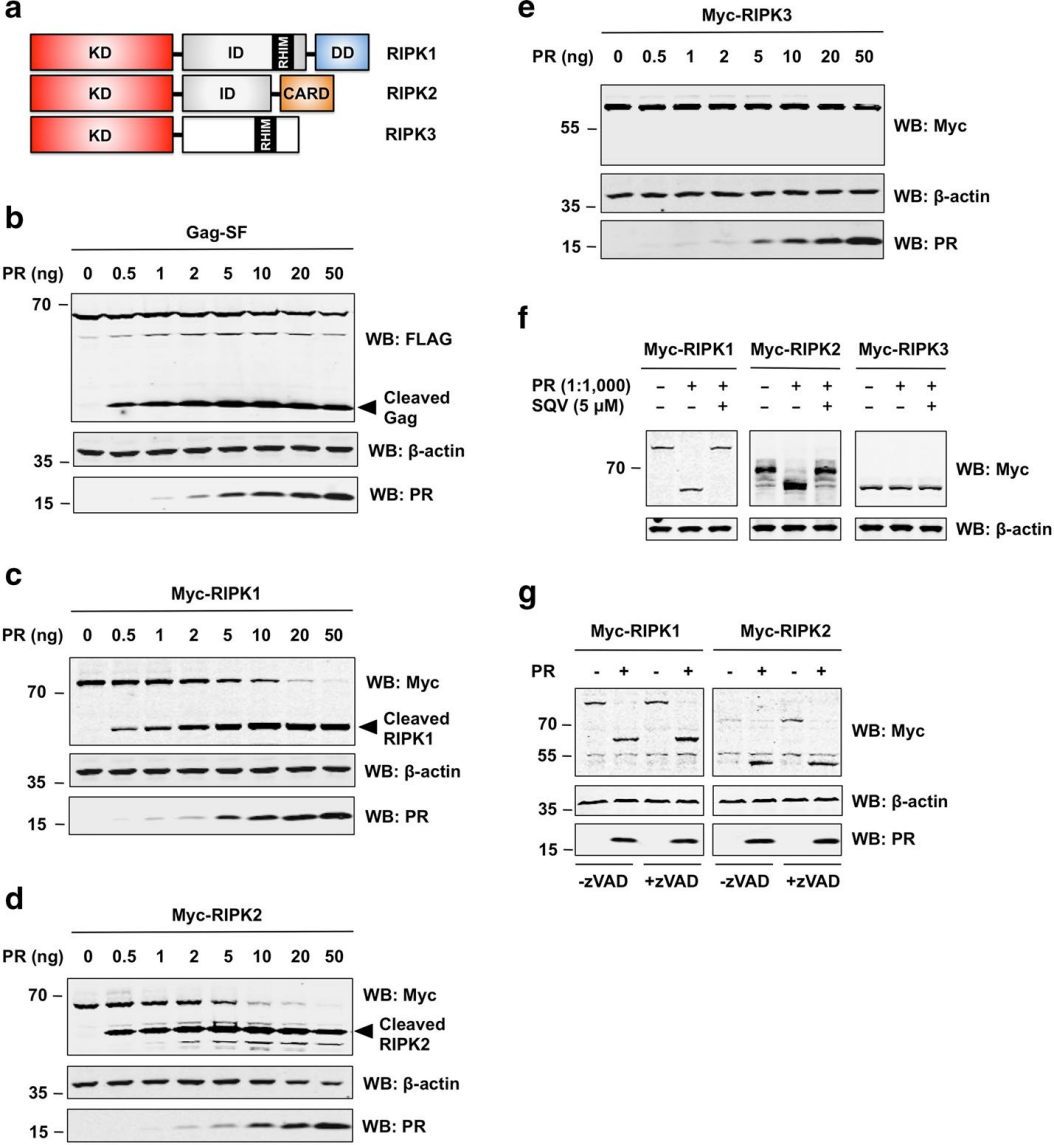
HIV-1 PR cleaves RIPK1 and RIPK2. **a** Domain architecture of RIPK family members. *CARD* caspase activation and recruitment domain, *DD* death domain, *ID* intermediate domain, *KD* kinase domain, *RHIM* RIP homotypic interaction motif. Illustration adopted from Festjens et al. [47]. **b**–**e** HEK293T cells were transfected with plasmids encoding Gag-SF (**b**), or Myc-tagged RIPK1 (**c**), RIPK2 (**d**), and RIPK3 (**e**) along with increasing amounts of plasmid encoding HIV-1 PR. After 24 h, cells were collected in lysis buffer and samples were subjected to SDS-PAGE and WB analysis. Proteins were revealed using antibodies against FLAG (for Gag), c-Myc (for RIPKs), β-actin, or HIV-1 PR. (F) HIV-1 PR cleaves RIPK1 and RIPK2 in vitro. HEK293T cells were transfected with expression plasmids encoding Myc-tagged RIPK1, RIPK2 or RIPK3. Cell lysates were prepared 24 h after transfection and incubated with recombinant HIV-1 PR (at a weight-to-weight ratio of 1000:1) in the absence or presence of SQV (5 μM). After 3 h of incubation at 37 °C, lysates were subjected to SDS-PAGE and WB. Proteins were revealed using antibodies against c-Myc or β-actin (loading control). **g** HIV-1 PR cleaves RIPK1 and RIPK2 in the absence of caspase activity. HEK293T cells were transfected with expression plasmids encoding Myc-tagged RIPK1 or RIPK2 with (+) or without (−) catalytically active HIV-1 PR in the absence or presence of pan-caspase inhibitor zVAD-fmk. Cells were lysed 24 h after transfection and total cell extracts were subjected to SDS-PAGE and WB. Proteins were revealed using antibodies described above

RIPK1, RIPK2 and RIPK3 are highly homologous to each other [47, 48] (Fig. 2a). We tested whether PR could cleave these three members of the RIPK family. Myc-tagged RIPK1, RIPK2 or RIPK3 were over-expressed in HEK293T cells and co-transfected with increasing amounts of a plasmid encoding PR. Although over-expression of catalytically active PR is cytotoxic, cells can tolerate transfection with smaller amounts of PR plasmid DNA; cell death is only observed upon transfection of larger amounts of PR. Transfection of HIV-Gag, a known target of PR, served as positive control (Fig. 2b).

We observed cleavage of RIPK1 (Fig. 2c) and RIPK2 (Fig. 2d) by PR under these experimental conditions. For both proteins, we observed the appearance of N-terminal cleavage products in the presence of minute amounts of PR plasmid DNA (0.5 ng/well). Complete cleavage of full length RIPK1 and RIPK2 was observed by co-transfection of only 20 ng PR expression plasmid. Even at higher concentrations of PR, no cleavage of β–actin was observed. Notably, the highly homologous RIPK3 protein was not cleaved by PR (Fig. 2e). In addition, we did not observe any cleavage of the upstream receptors NOD1 (Additional file 3: Figure S2A) and NOD2 (Additional file 3: Figure S2B), nor other crucial signaling proteins implicated in innate immune response to virus infection, including MAVS (Additional file 3: Figure S2C) and STING (Additional file 3: Figure S2D). Catalytic activity of PR was required for cleavage of RIPK1 and RIPK2 as no cleavage was observed upon transfection of catalytically inactive PR (D25N) (Additional file 3: Figure S2E). We also performed a detailed densitometric analysis of primary findings and bar graphs demonstrating comparative levels of full-length proteins can be found as supplemental material (Additional file 4: Figure S10).

We next asked whether cleavage of RIPK1 and RIPK2 by PR could be prevented by the PR inhibitor Saquinavir (SQV), the first HIV-1 PR inhibitor approved by the Food and Drug Administration (FDA). Indeed, we found that addition of SQV can completely abolished PR cleavage of RIPK1 and RIPK2. Dose–response experiments for RIPK2 show that complete inhibition was achieved by addition of 1 μM SQV, with partial inhibition observed at 0.1 μM (Additional file 3: Figure S2E).

As shown in Fig. 2f, HIV-1 PR can cleave RIPK1 and RIPK2 in vitro. Incubation of total cell extracts with recombinant HIV-1 PR at a weight-to-weight ratio of 1000 to 1 resulted in the cleavage of RIPK1 and RIPK2 and the loss of full-length proteins. Cleavage of RIPK1 and RIPK2 was completely prevented by addition of PR inhibitor SQV. Furthermore, PR did not cleave RIPK3 or β–actin in vitro.

It has previously been reported that PR cleaves and activates caspase-8 in vitro and during infection [16, 49, 50]. As RIPK family members are known substrates of active caspase-8 [51–53], it is possible that the observed cleavage of RIPK1 and RIPK2 could actually be due to caspase-8 activation by PR. We found that caspase-8 was processed by PR although to a much lesser extend than RIPK1 or RIPK2 (Additional file 5: Figure S3). However, inhibition of caspase activity by the pan-caspase inhibitor zVAD-fmk did not affect RIPK1 or RIPK2 cleavage by PR. We confirmed that zVAD-fmk was active in protecting cells from caspase-8-induced apoptosis (data not shown). Nevertheless, PR processed RIPK1 and RIPK2 equally in the absence or presence of zVAD-fmk (Fig. 2g). Therefore, RIPK1 and RIPK2 processing by HIV-1 PR is direct and does not dependent on the activation of caspases.

Taken together, our results demonstrate that PR cleaves RIPK1 and RIPK2 with high specificity.

### Endogenous RIPK1 is cleaved by HIV-1 PR

**Fig. 3:**
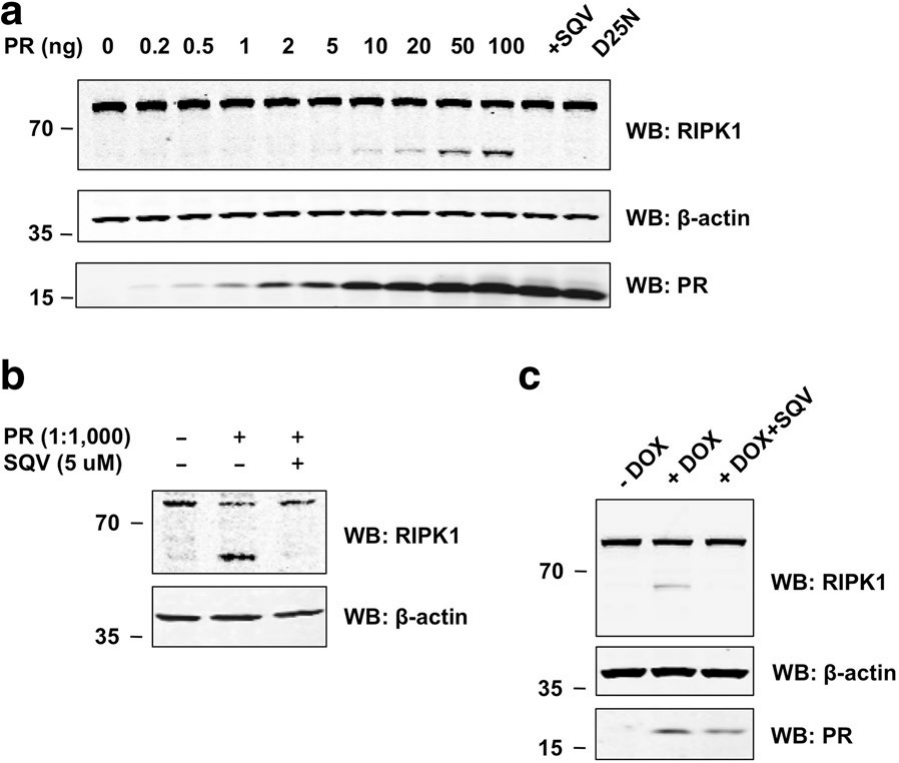
HIV-1 PR cleaves endogenous RIPK1. **a** HEK293T cells were transfected with empty vector (as control) or increasing amounts of an expression plasmid encoding catalytically active HIV-1 PR. Addition of SQV (5 μM) or transfection of catalytically inactive HIV PR D25 N served as negative controls. Cells were lysed 24 h after transfection and total cell extracts were subjected to SDS-PAGE and WB. Proteins were revealed using antibodies against RIKP1 (rabbit monoclonal antibody from Cell Signaling), β-actin, or HIV-1 PR. **b** HIV-1 PR cleaves endogenous RIPK1 in vitro. Jurkat cell lysates were incubated with recombinant HIV-1 PR (at a ratio of 1000:1) in the absence or presence of SQV (5 μM). After 3 h of incubation at 37 °C, lysates were subjected to SDS-PAGE and Western blotting (WB). Proteins were revealed using antibodies described above. **c** Jurkat cells with stably integrated Tet-inducible vector encoding catalytically active HIV-1 PR were cultured without (-DOX) or with doxycycline (+DOX, 1 μg/ml) in the absence or presence of SQV (5 μM). Total cell lysates were prepared 24 h after treatment and subjected to SDS-PAGE and WB. Proteins were revealed using antibodies as described above

Having demonstrated that PR can cleave over-expressed RIPK1 and RIPK2, we next investigated if PR can cleave endogenously expressed proteins. Antibody selection proved critical for detecting endogenous RIPK cleavage products. A rabbit monoclonal antibody raised against the N-terminus of RIPK1 (Cell Signaling) detected full length RIPK1 and the N-terminal cleavage product of both over-expressed and endogenous RIPK1 (Fig. 3a; Additional file 6: Figure S4A). In contrast, a mouse monoclonal antibody raised against the C-terminal of RIPK1 (BD Transduction Lab) failed to detect any RIPK1 cleavage products (Additional file 6: Figure S4A). Similar to our results for over-expressed proteins, cleavage of endogenous RIPK1 was absent in cells transfected with catalytically inactive PR or when treated with SQV (Fig. 3a). Furthermore, endogenous RIPK1 was processed in Jurkat cell lysates by recombinant PR in vitro (Fig. 3b).

Although we were able to detect cleavage of endogenous RIPK1, we have not yet been able to detect cleavage products of endogenous RIPK2 due to the lack of a suitable antibody. Furthermore, we have not been able to detect C-terminal cleavage products for either over-expressed or endogenous RIPK1. We ruled out that stability of respective cleavage products could be affected by the absence or presence of an epitope tag. PR cleaved N- and C-terminally tagged versions of RIPK1 at similar efficiency (Additional file 6: Figure S4B).

To extend our findings to a second cell line, we generated Jurkat cell lines with doxycyline-(DOX) inducible expression of catalytically active or inactive PR. Since leakiness of DOX systems is a known issue, we validated the “tightness” of the system by introducing a firefly luciferase reporter gene. Luciferase activity was undetectable (comparable to background levels) in non-induced cells (Additional file 7: Figure S5A). Furthermore, immunoblot analysis of cell lysates revealed undetectable levels of PR expression in non-induced cells. Induction of PR expression with DOX resulted in the production of cleaved RIPK1 (Fig. 3c). Treating these cells with SQV prevented this cleavage. Time-course studies showed that cleaved RIPK1 appeared after 8 h of DOX incubation, with a maximum after 24 h (Additional file 7: Figure S5B). Addition of SQV prevented the cleavage of endogenous RIPK1 at all times assayed. Dose–response experiments showed that elevated concentrations of DOX led to increased levels of cleaved RIPK1 in cells expressing catalytically active, but not inactive PR. (Additional file 7: Figure S5C). The second band observed in Figure S5B corresponds to a non-specific band and the differences compared to Fig. 3c can be attributed to batch-to-batch variations of the rabbit RIPK1 antibody. To further demonstrate that the additional band is indeed due to unspecific binding of the primary antibody (and not an additional RIPK1 cleavage product) we repeated the experiment in RIPK1 deficient Jurkat cells. Although no bands for full-length or cleaved RIPK1 were detected in lysates of *RIPK1*^−/−^ Jurkat cells, the respective unspecific band was still visible (Additional file 7: Figure S5D).

We also tested whether PR mutations, which occur in patients with discordant responses to antiretroviral therapy, differ in their ability to cleave RIPK1. We tested two discordance-associated mutations (DAMs), I54 V and V82A, which were previously shown to reduce the ability of PR to cleave caspase 8 [54]. Non-DAMs K20R, L63P, D30N and L90M served as negative controls. The active site dead D25G mutation served as positive control. With the exception of D25G (positive control), we did, however, not observe significant differences in RIPK1 cleavage for any of the PR mutations tested (Additional file 8: Figure S6). This indicates that differential processing of RIPK1 likely does not contribute to the discordant responses observed in patients carrying the I54 V or V82A mutations in PR.

Taken together, our results provide multiple lines of evidence that PR can cleave endogenous RIPK1.

### Identification of the PR cleavage site in RIPK1

**Fig. 4:**
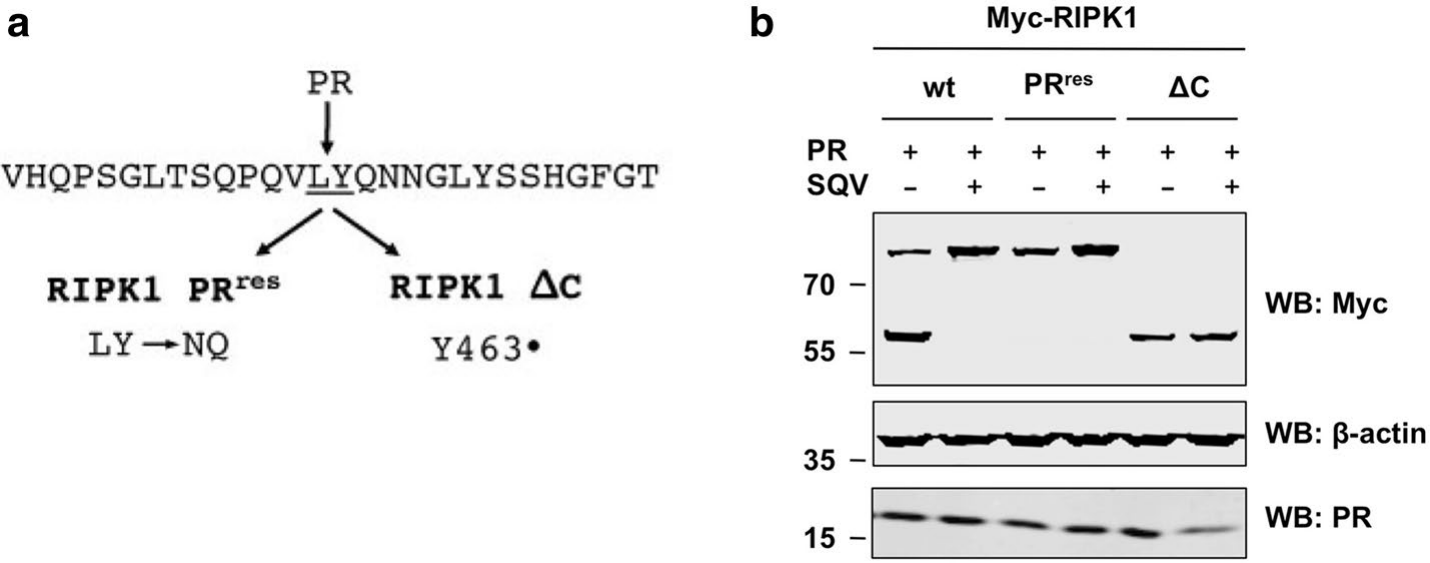
Identification of the PR cleavage site in RIPK1. **a** Primary sequence of human RIPK1 (aa 449–476 according to NCBI Reference Sequence NP_003795.2) with PR cleavage site between residues 462 and 463. Indicated point mutations were introduced by Quikchange mutatgenesis. **b** HEK293T cells were transfected with expression plasmids encoding wild-type (wt) RIPK1, RIPK1 with a double-mutation in the PR cleavage site (RIPK1 PR^res^), or RIPK1 with a non-sense mutation in the PR cleavage site (RIPK1 ΔC), respectively, along with catalytically active HIV PR (5 ng/well) in the absence (−) or presence (+) of SQV (5 μM). Cell lysates were subjected to SDS-PAGE and immunoblotting (WB). Proteins were revealed using antibodies against c-Myc, β-actin, or HIV-1 PR

We next used mass spectrometry to identify the precise cleavage site in RIPK1. To do so, we co-expressed Myc-tagged RIPK1 and PR in HEK293T cells. The N-terminal fragment of cleaved RIPK1 was purified from total cell extracts by immunoprecipitation with an anti-Myc antibody and purified complexes were subjected to SDS-PAGE analysis. A prominent band corresponding to the N-terminal cleavage product was submitted to mass spectrometry (Additional file 9: Figure S7A). Analysis revealed the presence of a non-tryptic peptide, corresponding to amino acids 436–462 of human RIPK1. This indicates that the cleavage site is located in the ID region of RIPK1 (Fig. 4a).

To experimentally confirm the cleavage site, we made two mutants. A double mutation of positions 462–463 from LY to NQ rendered RIPK1 resistant to PR cleavage (Fig. 4b). Introduction of a stop codon at position 463 resulted in production of a truncated version of RIPK1 (RIPK1 ΔC) that migrates at the exact same molecular size as the N-terminal RIPK1 cleavage product. Both of these findings suggest that the cleavage site is between L462 and Y463 (Fig. 4b).

Upon transfection of higher amounts of the PR expression construct (>10 ng per 1 × 10^5^ cells), we observed the appearance of a RIPK1 cleavage product, which migrated at a higher molecular weight. This indicates the presence of a secondary PR recognition site in RIPK1, which can serve, though much less efficiently, as an alternative cleavage site once the primary cleavage site is unavailable (Additional file 9: Figure S7B).

### RIPK1 and RIPK2 are cleaved during virus infection

**Fig. 5:**
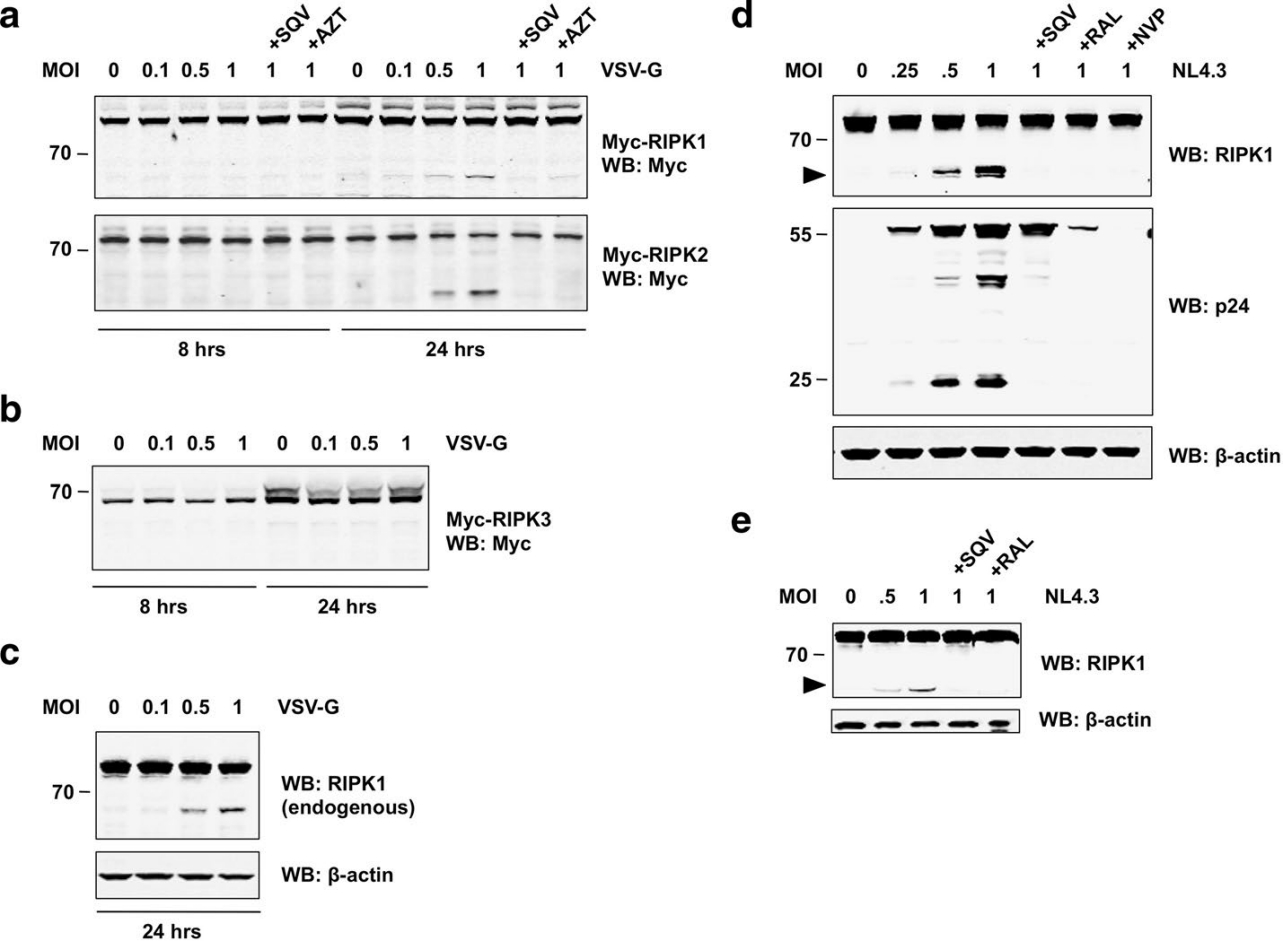
RIPK1 and RIPK2 are cleaved during HIV-1 infection. **a**, **b** HEK293T were transiently transfected with expression plasmids (0.5 μg/well) encoding Myc-tagged RIPK1, RIPK2, or RIPK3, respectively. After 24 h, cells were infected with different MOIs of VSV-G pseudotyped HIV-1 NL4.3 in the absence or presence of SQV (5 μM) or AZT (10 μM). Cell lysates were prepared 8 or 24 h after infection and subjected to SDS-PAGE and immunoblotting (WB). Proteins were revealed using anti-Myc antibody. **c**, **e** Endogenous RIPK1 is cleaved during HIV-1 infection. **c** HEK293T were infected with different MOIs of VSV-G pseudotyped HIV-1 NL4.3. Cell lysates were prepared 24 h after infection and subjected to SDS-PAGE and immunoblotting (WB). Proteins were revealed using antibodies against RIPK1 (rabbit monoclonal antibody from Cell Signaling) or β-actin. **d** Sup-T1 cells or **e** primary activated CD4^+^ T cells were infected with increasing MOIs of replication-competent HIV-1 NL4.3. Cells were cultured with or without SQV (5 μM), Raltegravir (RAL), or Nevirapine (NVP), respectively. Cell lysates were prepared 48 h after infection and subjected to SDS-PAGE and WB. Proteins were revealed using antibodies against RIKP1 (rabbit monoclonal antibody from Cell Signaling), p24, or β-actin

Having established that ectopically expressed or purified HIV-1 PR can cleave RIPK1 and RIPK2 in various cell types, we next asked whether RIPK1 and RIPK2 are cleaved during HIV-1 infection. First, we over-expressed Myc-tagged versions of RIPK1, -2, and -3 in HEK293T cells and infected with different MOIs of VSV-G-pseudotyped HIV-1 NL4.3. N-terminal cleavage products appeared 24 h after infection showing that RIPK1 and RIPK2 are cleaved during virus infection (Fig. 5a). As expected, addition of the PR inhibitor SQV completely blocked this cleavage. Moreover, treatment with AZT (an RT inhibitor) also inhibited RIPK1 and RIPK2 cleavage suggesting that integration and transcription of proviral genes are required for RIPK processing to occur. Importantly, RIPK3 was not cleaved under these conditions, demonstrating the specificity of our findings (Fig. 5b). We also quantified the levels of full-length and cleaved proteins and a comprehensive densitometric analysis of the band intensities in Fig. 5 can be found as supplemental material (Additional file 10: Figure S11).

Next, we tested whether endogenous proteins are processed during viral infection. Using non-transfected cells and a rabbit monoclonal antibody against RIPK1, we observed cleavage of endogenous RIPK1 in HEK293T cells infected with VSV-G-pseudotyped HIV-1 (Fig. 5c).

To validate our findings in a cell type with greater physiological relevance, we infected Sup-T1 cells, a T cell line, with replication-competent HIV-1 NL4.3. Forty-eight hours after infection, we observed cleavage of RIPK1, which again could be inhibited with SQV suggesting that PR plays a key role in this event (Fig. 5d). The amount of cleaved RIPK1 correlated with the appearance of processed p24-Gag in the cell extracts. Importantly, interfering with the viral life cycle at different stages by the addition of specific inhibitors against reverse transcriptase (Nevirapine, NVP) or integrase (Raltegravir, RAL), completely prevented RIPK1 cleavage (Fig. 5d). We have also observed very similar results in CEM cells, another T cell line (Additional file 11: Figure S8). These data suggest that it is the PR synthesized late in the viral life cycle and not the incoming PR that is responsible for processing of RIPK1.

We have also confirmed these findings in primary CD4^+^ T cells infected with replication-competent HIV-1. Consistent with our previous findings, endogenous RIPK1 was cleaved in infected primary cells; this event was sensitive to treatment with inhibitors against PR or integrase (Fig. 5e).

Taken together, our results demonstrate cleavage of RIPK1 in T cell lines and primary CD4^+^ T cells during HIV-1 infection. This cleavage is observed at later time points of infection and is prevented not only by direct inhibition of PR, but also by addition of other inhibitors that interfere at early stages of the viral life cycle.

### RIPK1 processing occurs in the absence of “incoming” PR

To further address the source of PR responsible for the observed cleavage of RIPK1—incoming viral particles or newly produced proteins in the infected cell—we transfected HEK293T cells with proviral plasmids routinely used for production of lentiviral stocks, pNL4.3-Luc-Env^−^ (NIH AIDS Research Program, [55]) or psPAX2 [56], in the absence of an Env-encoding plasmid. This ensures that released virions could not re-infect cells. Cleaved RIPK1 appeared 20 h after transfection of lentiviral plasmids (Additional file 12: Figure S9). The cleavage became more pronounced 40 h after tranfection and depended on the catalytic activity of PR. Addition of SQV at the time of transfection completely abolished RIPK1 processing. Cells transfected with psPAX2, which induces elevated expression of proviral genes from a CMV promoter, displayed more RIPK1 cleavage compared to cells transfected with pNL4.3-Luc-Env^−^, which features LTR-driven expression of proviral genes.

Although we cannot exclude the possibility that PR delivered by virions at the time of infection can cleave RIPK1, our results indicate that the majority of RIPK1 cleavage occurs due to post-integration expression of proviral genes. This explains why we did not detect cleaved RIPK1 at early time points or when the later stages of infection were blocked either with RT or integrase inhibitors.

### PR cleavage disrupts RIPK1 function

Having established that PR cleaves RIPK1 and RIPK2 during HIV-1 infection, we sought to determine whether cleavage of RIPKs deregulated host processes that may impact virus replication. For example, RIPK1 is known to play a critical role in TNF signaling via NF-κB [57, 58]. However, when NF-κB signaling is impaired, cleavage of RIPK1 has been reported to play a key role in controlling apoptosis. RIPK1 can also initiate necroptosis by interacting with RIPK3 [30, 59]. We therefore conducted experiments to assess the effects of PR cleavage on these activities of RIPK1.

**Fig. 6:**
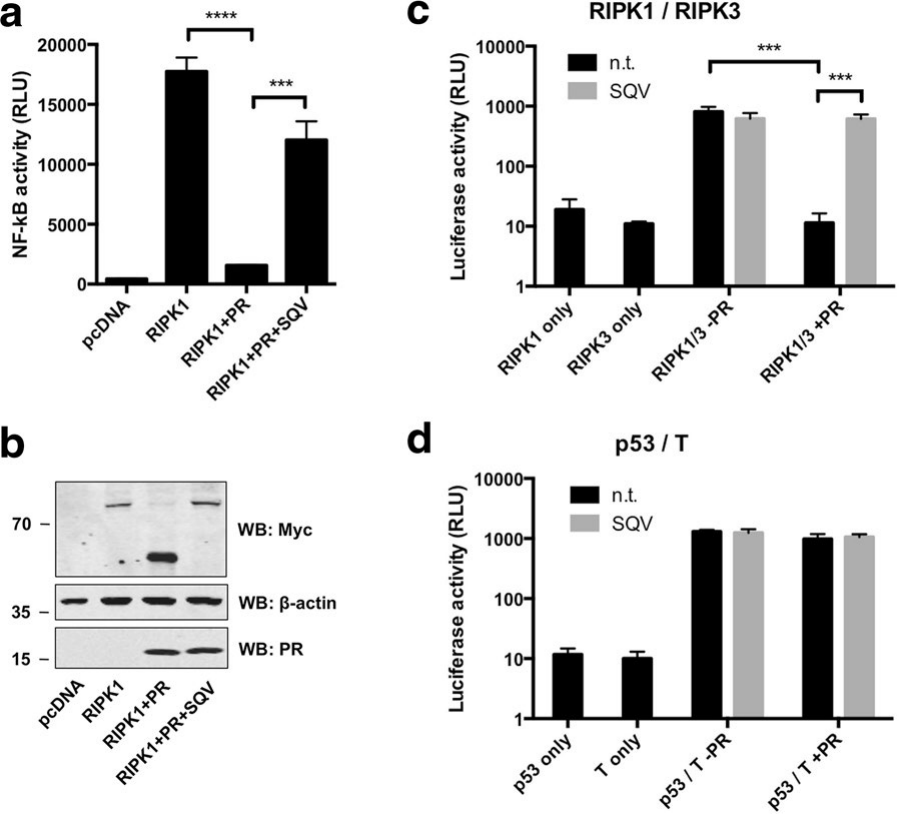
Processing by HIV-1 PR disrupts RIPK1 function. **a** HEK293T cells harboring a stable NF-κB luciferase reporter were transfected by lipofection with an expression plasmid encoding RIPK1, or empty pcDNA. Cells were co-transfected with or without PR (20 ng/well), then cultured with or without SQV. After 24 h, cells were lysed by addition of Steady-Glo^R^ (Promega) and luciferase activity was measured using a standard plate luminometer. **b** Protein levels in **a** were analyzed using standard SDS-PAGE/Western blotting techniques. Proteins were revealed using antibodies against c-Myc, β-actin, or HIV-1 PR. Results are from a single experiment and are representative of at least three separate experiments. **c**, **d** Mammalian 2-hybrid system. **c** HEK293T cells were transfected with plasmids encoding RIPK1 fused to the GAL4 DNA-binding domain (RIPK1 only), RIPK3 fused to the GAL4 transcription activation domain (RIPK3 only), or a combination thereof (RIPK1/3). Cells were co-transfected with (+) or without (−) PR in the absence (n.t.) or presence (+) of SQV. **d** HEK293T cells were transfected with plasmids encoding p53 fused to the GAL4 DNA-binding domain (p53 only), large T antigen fused to the GAL4 transcription activation domain (T only), or a combination thereof (p53/T). Values represent mean ± standard deviation of triplicate cultures (mean ± SD, n = 3). Statistical significance was determined by unpaired t-tests, ****P ≤ 0.0001, ***P ≤ 0.001

First, we examined if PR cleavage can diminish the ability of RIPK1 to activate NF-κB. Over-expression of RIPK1 in HEK293T cells leads to activation of NF-κB, as indicated by the induction of an NF-κB-driven luciferase reporter gene. Co-expression of PR in this context resulted in a loss of luciferase activity that was completely restored by treatment with PR inhibitor SQV (Fig. 6a). Western blot analysis revealed that under these experimental conditions levels of full-length RIPK1 were significantly reduced and thus RIPK1 function was lost (Fig. 6b).

We also tested if PR cleavage can disrupt the interaction of RIPK1 with RIPK3. For these experiments, we employed a mammalian 2-hybrid system to test whether PR could affect the interaction of RIPK1 with RIPK3. In this approach, binding of the two proteins drives the expression of a luciferase gene. As expected, RIPK1 and RIPK3 demonstrated a robust interaction (Fig. 6c). Co-expression of increasing amounts of PR resulted in complete loss of luciferase activity, indicating disruption of the RIPK1/RIPK3 interaction. Loss of luciferase activity was completely rescued by the addition of SQV (Fig. 6c). Importantly, the interaction of two unrelated proteins (p53 and large T antigen) was not affected by co-expression of PR (Fig. 6d). Taken together, these results demonstrate that catalytic processing by PR disrupts critical RIPK1 functions.

## Discussion

We have found that RIPK1 and RIPK2 are novel host substrates of HIV-1 PR. Cleavage of RIPK1 and RIPK2 was observed in human cells expressing PR and when infected with HIV-1. A small molecule inhibitor of PR completely blocked cleavage of RIPK1 and RIPK2. The PR cleavage site in RIPK1 was identified by mass-spectrometry and experimentally confirmed by showing that a mutation designed to disrupt the cleavage site inhibited processing. Over-expression of PR did not lead to promiscuous, non-specific cleavage of host proteins, as shown for example by comparisons with RIPK3.

HIV-1 PR is known to target host proteins. This activity likely represents an important viral strategy to counter host restriction factors. However, the large numbers of putative host cell substrates found in recent proteomics screens [18, 19] raises the question of how specific and how relevant PR-mediated cleavage of any given factor actually is. Over-expression of PR in vivo or high concentrations of PR in vitro results in the proteolytic cleavage of many substrates. Studies under more physiologically relevant conditions or actual viral infections result in selective cleavage of fewer targets. It is thus not surprising that the number of factors found cleaved during viral infection will likely be far smaller than the total number of factors cleaved in vitro (Additional file 1: Table S1). Although a number of potential host-encoded substrates have been reported for HIV-1, a limited subset of host proteins has been demonstrated to be cleaved in primary cell types targeted during the course of natural infection. Data presented here demonstrating cleavage of RIP kinases in CD4^+^ T cells challenged with HIV-1 indicates that these proteins belong to this fraction of bona fide HIV-1 protease substrates.

In fact, RIPK1 and RIPK2 appear to be excellent substrates for PR, based on the efficiency of their cleavage in cells expressing minute amounts of PR. The cleavage site we identified is consistent with predicted PR cleavage sites [60]. Defining a “consensus PR cleavage site” though is difficult, as PR is capable of cleaving a wide variety of amino acid sequences. The efficiency of by which PR cleaves multiple target sites in Gag and Gag-Pol varies considerably, and its different target sites display limited primary sequence homology. This is consistent with the hypothesis that substrate shape rather than primary sequence is important for substrate recognition by PR [61].

We have also sought to determine when in the viral life-cycle does RIPK1 and RIPK2 get targeted for cleavage. It is important thus to know where and when PR becomes catalytically detectable within infected cells. It is well know that PR activity is critical for proteolytic processing of viral precursor proteins thereby ensuing proper structural arrangements in newly released viruses. It is not entirely clear though how much catalytically active PR is present in HIV-1 infected cells. The mechanism by which PR is activated during viral assembly is not fully understood. PR is inactive within the Gag-Pro-Pol polyprotein because the mature active enzyme is a dimer. Gag trafficking probably helps to regulate PR activation. Gag and Gag-Pol molecules arrive at the plasma membrane as oligomers, and polymerize into membrane-bound assembly sites where Gag levels increase exponentially [62, 63]. High concentrations of Gag and Gag-Pol precursors facilitate formation of transient dimeric “encounter” complexes that have enzymatic activity. Within these, autoprocessing and formation of stable PR dimers with full catalytic activity can ensue [12]. PR activity in the cytosolic fraction of infected cells has also been documented [13, 14, 49]. This intracellular PR activity may allow for proteolytic processing of host cell proteins. PR activity might also play a role in early stages of infection. It was reported that treatment with PR inhibitors reduces viral replication in a single cycle of infection presumably by altering the stability of unintegrated viral cDNA [64]. These data indicate that “incoming PR” can target host cell factors and counteract cellular restriction. While we cannot rule out that PR released during virus entry might target RIPK1 and RIPK2, our data suggest that RIPK1 and RIPK2 cleavage occurs at later stages of infection and depends on transcription of viral genes. Thus, we favor the interpretation that “de novo” activity of PR is responsible for RIPK1 and RIPK2 cleavage in HIV infected cells.

PR’s role in counteracting cellular immune mechanisms is just beginning to emerge. Our results show that PR cleavage disrupts the function of RIPK1. HIV-1 infection can elicit an innate immune response, resulting in the production of interferons (IFNs) or pro-inflammatory cytokines such as TNF-α. Known HIV-1 PAMPs include the viral capsid (CA) and viral nucleic acids such as the incoming RNA or the reverse-transcribed DNA [3, 65, 66]. To successfully establish a persistent infection, HIV-1 relies on a variety of countermeasures to suppress innate immune signaling.

Numerous HIV-mediated immune evasion strategies have been described [67–69]. The ability of HIV to counteract host defenses has been linked to the presence of accessory genes, such as Vpu and Vif [9, 70, 71]. Our data suggest another mechanism by which HIV may counteract host innate immune responses—namely by cleaving and inactivating RIPK1 and presumably (though not tested here) RIPK2. HIV seems to possess redundant mechanisms for ensuring suppression of the type I IFN pathway. As demonstrated by Solis et al., PR-mediated inhibition of RIG-I sensing contributes to viral suppression of the IFN response in macrophages [72]. Interestingly, Rajput et al. [53] demonstrated that cleavage of RIPK1 by caspase-8 inhibits type I IFN signaling downstream of the ribonucleic acid sensor RIG-I. In this context, caspase-8-mediated cleavage of RIPK1 converts RIPK1 from a signaling enhancer to a signaling inhibitor. Based on these observations, we wondered whether PR could mimic the role of caspase-8 in attenuating type I IFN signaling by directly cleaving RIPK1 during HIV-1 infection. Although preliminary experiments confirmed that over-expression of PR suppresses RIG-I-mediated type I IFN signaling, we were not able to find any evidence that this suppression was linked to the ability of PR to cleave RIPK1. Moreover, subsequent experiments indicated that PR-mediated inhibition of RIG-I activity was not affected by the absence or presence of functional RIPK1 (data not shown).

However, RIPK1 integrates multiple signals from the innate immune system, including those generated in response to inflammatory cytokines and to detection of pathogens. Of particular interest is the recruitment of RIPK1 to various nucleic acid-sensing platforms. For example, RIPK1 was shown to promote IRF3 and NF-κB activation downstream of Toll-like receptors (TLRs) and the cytosolic DNA sensor DAI [27, 73]. RIPK1 interactions with TLR3 or DAI are mediated by RHIM and play important roles during virus infection. PR cleavage therefore could impact numerous RIPK1 functions beyond the traditional role associated with its participation in TNFR1 signaling. Cellular responses controlled by RIPK1 are diverse, and include the activation of MAP kinases and NF-κB as well as induction of both apoptotic and necrotic cell death [74].

RIPK1 interacts with RIPK3 through its RHIM domain. The RIPK1/RIPK3 interaction is critical for the induction of necroptosis, an inflammatory form of cell death, often observed upon DNA virus infection. Necroptosis leads to release of “danger signals” (endogenous PAMPs) that activate the immune response. Viruses try to limit necroptosis to avoid activation of the immune system. Some DNA viruses inhibit RIPK1-mediated induction of necroptosis by encoding RHIM-homology proteins [75]. Our data suggest that PR cleavage can disrupt RHIM-mediated interaction of RIPK1 and RIPK3. Thus, another possibility is, during HIV infection, PR activity may mirror the role of viral RHIM-homology proteins in suppressing the induction of necroptosis.

Whereas the role of RIPK2 in defense to bacterial infection is well established, evidence for RIPK2 function in response to viral infection is just beginning to emerge. RIPK2 appears to play a protective role during influenza A virus infection. It does so, interestingly, in a kinase-dependent manner by suppressing activation of the NLRP3 inflammasome via a mitochondrial pathway [43]. In response to certain DNA viruses, RIPK2 may also promote the induction of IFN downstream of pattern-recognition receptor NOD2 [76]. The importance of RIPKs in combating viral infections is further highlighted by the fact that both RIPK1 and RIPK2 are known ISGs [77].

Interestingly, RIPK family members also have known connections to NLRs. RIPK1 function was shown do be essential for activation of the NLRP3 inflammasome upon infection with RNA viruses, such as Sendai virus or Influenza A [41]. Infection with RNA viruses triggers the formation of a RIPK1/RIPK3 complex, and inhibition of RIPK1 kinase function blocked viral-induced activation of the inflammasome. Since different reports suggest that NLRs can be activated during HIV infection, the connection between RIPKs and NLRs is intriguing. HIV-1 can trigger NLRP3 inflammasome activation and release of IL-1β [78–81]. It has been suggested that gp120 functions as the PAMP for inflammasome activation in HIV-infected cells since VSV-G pseudotyped viruses do not elicit a response [81]. Whether HIV-1 infection leads to the formation of a RIPK1/RIPK3 complex that could promote NLRP3 inflammasome activation remains to be elucidated.

Ectopic expression of RIPK1 and PR results in levels of full-length RIPK1 that are significantly reduced (Fig. 2c). While we still observed cleavage of endogenous RIPK1 after expression of PR or HIV-1 challenge, we were unable to detect a corresponding reduction of full-length RIPK1 (Figs. 3a, 5). While the basis of this difference is currently unclear, we hypothesize that only a fraction of endogenous RIPK1 may be targeted by PR for cleavage, possibly dependent on unique subcellular localization, higher-order complex formation, or post-translational modifications. We further speculate that the kinetics of these event(s) may be altered with RIPK1 species that are newly synthesized after ectopic expression thereby making it more accessible for PR cleavage. Alternatively, cleavage of endogenous RIPK1 may trigger a feedback circuit that increases synthesis of RIPK1. These results suggest that the cleaved species of RIPK1 may possess dominant negative activity that impedes critical, yet unknown, cellular functions that facilitate HIV-1 replication and/or pathogenesis.

Proteolytic control of RIPK1-mediated functions has been extensively described in distinct contexts, and there are various examples where cleavage of RIPK1 impacts important biological processes despite modest changes in the levels of full-length protein. RIPK1 can be cleaved by cellular proteases such as caspase-8 [51] or by cathepsin B [82]. Activation of death receptors (TNF-R1, Fas, DR3, DR4 or DR5) promotes the caspase-mediated cleavage of RIPK1 resulting in the generation of biologically active fragments with distinct functions that modulate NF-κB-mediated cell survival or death [51]. TNF signaling and concomitant inhibition of caspases by treatment with pan-caspase-inhibitor zVAD leads to increased phosphorylation and decreased cleavage of RIPK1, leading to TNF-induced necroptosis. Similarly, TLR stimulation or treatment with IFNs in the presence of zVAD results in RIPK1 phosphorylation and induction of necroptosis [83–85]. Moreover, RIPK1-mediated RIG-I-induced IRF3 activation appears to be controlled by recruitment of caspase-8 to the RIG-I-MAVS complex, as cells lacking caspase-8 displayed dramatically elevated IRF3 activation in response to Sendai virus or transfected dsRNA [53]. A common phenomenon between our study and the reports described above is the extent to which RIPK1 cleavage is observed. In all cases, levels of full-length RIPK1 remain relatively unchanged, but cleaved products persist within the cell and the proteolytic processing of RIPK1 is clearly of physiological relevance [51, 53, 82]. During HIV infection, the N-terminal RIPK1 cleavage product appears to be stable in the cell and it comprises the RIPK1 kinase domain (KD). However, elucidating the basis of the observed discrepancy between in vitro and in vivo RIPK1 cleave by PR, as well as understanding the impact of this event on RIPK1 function, will be critical in determining the influence of this event on HIV-1 replication and pathogenesis.

## Conclusions

Our results show that RIPK1 and RIPK2 are *bona fide* substrates of HIV-1 PR. Further investigation of the functional implications of this activity on potentially a diversity of innate and adaptive immune responses will provide critical insight towards strategies by which the virus manipulates host molecular circuits to promote pathogenesis. RIP kinase family members control a large number of cellular processes and are regulated through proteolytic cleavage. HIV-1 PR has likely evolved to mimic these cellular activities to establish a favorable environment for viral replication. The diverse roles of RIPK family members in response to other viruses are well documented. PR-mediated proteolytic modification of RIPK1 and RIPK2 therefore likely represents an important strategy of HIV-1 to counter cellular restriction and modulate the host response to infection.

## Methods

### Reagents

Doxycycline, Saquinavir, Azidodeoxythymidine (AZT), Nevirapine, and Raltegravir were purchased from Santa Cruz Biotechnology; human TNF-α was from Cell Signaling Technology; z-VAD-fmk was from R&D Systems; Birinapant was from BioVision; recombinant HIV-1 protease was from Prospec Tany TechnoGene; cOmplete Protease Inhibitor tablets were from Roche Diagnostics; all primers used for PCR and qRT-PCR were from IDT.

### Cell lines and tissue culture

HEK 293T and Jurkat cells were purchased from ATCC. Sup-T1 and CEM-SS cells were obtained from the NIH AIDS Reagent Program. HEK 293T cells were cultured in Dulbecco’s Modified Eagle Medium (DMEM) supplemented with 10 % FBS (Thermo Scientific), l-glutamine and penicillin/streptomycin. Jurkat, Sup-T1, and CEM-SS cells were cultured in Roswell Park Memorial Institute medium (RPMI-1640), supplemented with 10 % FBS, l-glutamine and penicillin/streptomycin. Stable Jurkat cell lines with Tet-inducible expression of HIV-1 PR (Jurkat Tet-On-PR) or catalytically inactive HIV-1 PR D25N (Jurkat Tet-On-PR D25 N) were generated following the manufacturer’s instructions (Lenti-X™ Tet-On^R^ Advanced Inducible Expression System, Clontech Laboratories). Stable transformants were selected using 200 μg/ml G418 and 1 μg/ml puromycin.

### Expression vectors

Coding regions of human RIPK1 (GenBank^R^ accession number NM_003804), RIPK2 (NM_003821), and RIPK3 (NM_006871) were PCR amplified and cloned into pcDNA3 carrying a N-terminal Myc-tag using standard molecular biology techniques. Identities of ORFs were sequence verified by 5′ and 3′ end sequencing. The plasmid encoding human RIPK1 was further used for creation of the RIPK1 PR^res^ and RIPK1 ΔC mutants, using the QuikChange II Site-Directed Mutagenesis Kit (Agilent Technologies). To generate RIPK1 PR^res^, we employed the site-directed mutagenesis and oligonucleotides that altered Leu-Tyr at position 462 and 463 (according to NCBI Reference Sequence NP_003795.2) to Asn-Gln. The RIPK1 ΔC expression construct was similarly generated using site-directed mutagenesis and oligonucleotides that introduced a stop codon at position 463. For mammalian two-hybrid analysis, coding sequences of human RIPK1 and RIPK3 were subcloned into pCMV-BD and pCMV-AD (Stratagene). Plasmids encoding codon-optimized versions of HIV-1 ORFs, including HIV-1 PR and PR (D25N), were kindly provided by Dr. Nevan Krogan [19]. Plasmids encoding discordance-associated mutations of HIV-1 PR were kindly provided by Dr. Andrew Badley [54]. Plasmids encoding NOD1, NOD2, MAVS, and STING were property of our lab and were all in CMV promoter-driven vectors.

### Affinity purifications and immunoblot analysis

For APs, 1 × 10^6^ HEK 293T cells were seeded in 6-well plates and the next day transfected with 1–2 μg plasmid using Lipofectamine^R^ 2000 (Life Technologies). Twenty-four hours after transfection, cells were detached and washed with ice-cold PBS. Cells were lysed in 1 ml ice-cold lysis buffer (50 mM Tris pH 7.4, 150 mM NaCl, 0.5 % Nonidet P40, supplemented with cOmplete Protease Inhibitor mix). Cell lysates were cleared by centrifugation at 14,000×*g* for 15 min. Cleared lysates were incubated with 5 μL Dynabeads M-280 Streptavidin for 2 h at 4 °C with rotation. The beads were washed 5× with ice-cold lysis buffer. Proteins were eluted by boiling in SDS-sample buffer at 95 °C for 5 min. APs or whole cell lysates were resolved on Novex^R^ 4–20 % Tris–Glycine gels (Life Technologies), transferred to a nitrocellulose membrane using the Trans-Blot^R^ Turbo™ system (BioRad), blocked with 5 % milk/TBST, probed overnight at 4 °C with primary antibodies, then incubated with fluorescent secondary antibodies for 1 h at room temperature. Proteins were detected and quantified using the Odyssey Infrared Imaging System (LI-COR Biosciences). The following primary antibodies were used: mouse monoclonal anti-c-Myc (Santa Cruz Biotechnology), mouse monoclonal anti-FLAG M2 (Sigma), mouse monoclonal anti-HA (Santa Cruz Biotechnology), mouse monoclonal anti-β-actin (Sigma), mouse monoclonal anti-RIP (BD Transduction Laboratories), rabbit monoclonal RIP (D94C12) XPR antibody (Cell Signaling Technology), mouse monoclonal anti-RIP2/RICK (BD Transduction Laboratories), rabbit polyclonal anti-RIP3 (Abcam), mouse monoclonal anti-p24 (MP Biomedicals), and mouse monoclonal anti-HIV-1 protease (Life Technologies). The secondary antibodies used were donkey (polyclonal) IRDye^R^ 680RD anti-Mouse IgG and donkey (polyclonal) Dye^R^ 800CW anti-rabbit IgG (LI-COR Biosciences).

### Protease cleavage studies

For protease cleavage studies, 5 × 10^5^ HEK 293T cells were seeded in 12-well plates and the next day cotransfected with plasmids encoding HIV-1 Gag (as positive control) or indicated human proteins (0.5 μg/well), along with increasing amounts of plasmid encoding HIV protease (PR). Addition of SQV (5 μM) or transfection of plasmid encoding catalytically inactive HIV protease (D25 N) served as negative controls. Twenty-four hours after transfection, cells were detached and washed with ice-cold PBS. Cell pellets were lysed by addition of SDS-sample buffer and incubation at 95 °C for 5 min. Total cell extracts (10 %) were subjected to SDS-PAGE and immunoblotting. For in vitro cleavage assays, HEK 293T or Jurkat cells were lysed in lysis buffer (50 mM Tris.Cl, pH 7.4, 150 mM NaCl, 0.5 % NP-40, without protease inhibitors). Cell lysates were cleared by centrifugation at 14,000×*g* for 15 min. Protein concentration was determined using the Bio-Rad protein assay kit (Bio-Rad Laboratories). Cleared lysates were incubated with recombinant HIV-1 protease (at a weight to weight ratio of 1000:1) at 37 °C for 3 h.

### Sample preparation for mass spectrometry

Coomassie stained gel bands were cut to 1 × 1 mm pieces and transferred to separate new ethanol rinsed eppendorff tube and were de-stained using 50 % ACN in 50 mM Ammonium Bicarbonate. Following vacuum drying, proteins were reduced and alkylated by final concentration of 5 mM DTT and 15 mM iodoacetamide prior to digestion by Trypsin at a final concentration of 25 ng/μl in 50 mM ammonium bicarbonate for 1 h on ice and additionally 16 h more at 37 °C using shaking incubator to assure complete digestion. Digested Tryptic peptides were extracted from gels and transferred to a new tube by following elution process; 100 μl of water added to the gels, sonicated 10 min in water bath and then flowed by 1 time in 5 % Formic acid in water and 4 time extraction by 50 % Acetonitrile in 5 % Formic Acid in water, once in 70 % acetonitrile and last in 100 % acetonitrile; all extracted peptides were pooled together and were vacuum dried and re-dissolved in 20 μl of 0.1 % TFA. Digested peptides were then concentrated and desalted using a Millipore C18 Zip Tip (Millipore). The eluent were then vacuum dried and re-dissolved in 24 μl of LC/MS loading buffer (2 % acetonitrile in 0.1 % formic acid in water).

### Mass spectrometry

Eight microlitre of tryptic digested samples were loaded to the automated Nano LC- LTQ MS/MS (Thermo Scientific, Waltham, MA, USA), using an Eksigent Nano 2D LC system, a switch valve, a C18 trap column (Agilent, Santa Clara, CA, USA), a Zorbax C18 peptide trap column (Agilent technologies), and a capillary reversed phased column (15 cm in length, 100 mm id) packed with 5 mm, Magic C18 AQ resin (Michrom) with an ADVANCE ESI source (Michrom) which is used to ionize the peptides as they elute from the RP column, using a linear gradient elution from buffer A (2 % acetonitrile in H2O plus 0.1 % formic acid) to 15 % buffer A plus 85 % buffer B (ACN plus 0.1 % formic acid) in 120 min. The LC/MS run was operated in the data dependent mode. Data on the four strongest ions above an intensity of 50 × 10^e4^ were collected with dynamic exclusion enabled and the collision energy set at 35 %). The MS/MS spectra were analyzed by Sorcerer Enterprise v.3.5 release (Sage-N Research Inc.) with SEQUEST algorithm as the search program for peptide/protein identification. SEQUEST was set up to search the target-decoy ipi.Human.v3.73 database containing protein sequences using trypsin for enzyme with the allowance of up to 2 missed cleavages, Semi Tryptic search and precursor mass tolerance of 1.5 amu. Differential search includes 16 Da for methonine oxidation and 57 Da for cysteines to account for carboxyamidomethylation in case of alkylation of cysteines. The search results were viewed, sorted, filtered, and statically analyzed by using comprehensive proteomics data analysis software, Peptide/Protein prophet v.4.02 (ISB).

### Isolation of human CD4^+^ T cells

Peripheral blood mononuclear cells (PBMC) were isolated by Ficoll density gradient centrifugation (Histopaque; Sigma) from anonymous healthy human donors (Scripps Normal Blood Donor Services, The Scripps Research Institute). CD4^+^ T cells were negatively selected using magnetic beads (CD4^+^ T cell isolation kit II; Miltenyi Biotec) as per the manufacturer’s instructions. CD4^+^ T cells were cultured in RPMI 1640 supplemented with 10 % FBS, l-glutamine, penicillin/streptomycin, and 20 units/ml interleukin-2 (NIH AIDS Reagent Program). Lymphocytes were activated with 1 μg/ml phytohemagglutinin-P (PHA) (Sigma) for 48 h.

### Preparation of virus stocks

The proviral plasmids pNL4.3 and pNL4.3-Luc-E^−^ were obtained from the NIH AIDS Reagent Program. Replication-competent HIV was generated by transfecting HEK 293T cells with the pNL4.3 proviral plasmid using Lipofectamine^R^ 2000. VSV-G pseudotyped NL4.3 for single-cycle infections was generated by cotransfection of HEK 293T cells with pNL43-Luc-E^−^ and pHCMV-G. Two days after transfection, supernatants were collected, filtered (0.45 μm), concentrated using Lenti-X™ concentrator (Clontech Laboratories), aliquoted, and stored at −80 °C. Physical titers of the viral stocks were determined using the AlphaLISA HIV p24 Kit (Perkin Elmer LAS).

### Virus infection

For detection of RIPK cleavage during single-round infection, HEK 293T were transiently transfected with RIPK1, RIPK2, or RIPK3 expression plasmids (0.5 μg/well). Twenty-four hours after transfection cells were infected with indicated MOIs of VSV-g pseudotyped HIV NL4.3 in the presence or absence SQV (5 μM) or AZT (10 μM). Cell lysates were prepared 8 and 24 h post infection. For detection of RIPK cleavage during spreading-infection, Sup-T1 or CEM-SS cells were infected with increasing MOIs of replication-competent HIV NL4.3. Cells were spin-occulated at 1200×*g* for 60 min. Afterwards, cells were either left non-treated or treated with SQV (5 μM), Raltegravir (5 μM), or Nevirapine (5 μM). Cell lysates were prepared 48 h post infection. Primary CD4^+^ T cells were PHA activated for 48 h before infection with HIV NL4.3 by spin occulation. Cell lysates were prepared 48 h post infection.

### Luciferase and cell viability measurement

For measurement of NF-kB activity, HEK 293T cells harboring a stable NF-κB luciferase reporter were transfected with an expression plasmid encoding RIPK1, or empty pcDNA using Lipofectamine 2000 (Life Technologies). Cells were co-transfected with or without PR, then cultured with or without SQV. For mammalian two-hybrid assays, HEK 293T cells were transfected with pCMV-BD, pCMV-AD, and luciferase reporter plasmids (Stratagene). Twenty-four hours after transfection, firefly luciferase activity was quantified with Britelite Plus (PerkinElmer). All experiments were performed in triplicate. Luciferase assays and cell viability assays were quantified by using the PHERAstar luminometer (BMG Labtech).

### Statistical analysis

Statistical analysis was performed using GraphPad Prism 6 software.

## Additional files

Additional file 1:
**Table S1.** Host cell substrates of HIV-1 PR. Known host cell substrates of HIV-1 PR were curated from the scientific literature and listed according to their publication date. Supporting evidence for each substrate is specified along with respective references to original publications. Additional file 2:
**Figure S1.** Endogenous RIPK2 specifically binds HIV-1 PR. Various SF-tagged HIV-proteins were expressed by transfection in HEK293T cells. After 24 hours, proteins were affinity purified from cleared cell lysates with Dynabeads Streptavidin. Affinity purifications (APs) or total lysates were subjected to SDS-PAGE and Western blotting (WB). Proteins were revealed using antibodies against RIPK2 (BD Transduction Laboratories), or FLAG (Sigma). Additional file 3:
**Figure S2.** HIV-1 PR does not cleave unrelated proteins. (A)-(D) HEK293T cells were transfected with plasmids encoding Myc-tagged NOD1 (A), and NOD2 (B), or FLAG-tagged MAVS (C), and STING (D) along with increasing amounts of plasmid encoding HIV-1 PR. After 24 hours, cells were collected in lysis buffer and samples were subjected to SDS-PAGE and WB analysis. Proteins were revealed using antibodies against c-Myc, FLAG, β-actin, or HIV-1 PR. (E) Dose-dependent inhibition of RIPK2 cleavage. HEK293T cells were transfected with Myc-RIPK2 in the absence (-) or presence (+) of catalytically active HIV PR (PR) or catalytically inactive HIV PR (D25N) (10 ng/well) and increasing concentrations of PR inhibitor SQV. Cell lysates were subjected to SDS-PAGE and immunoblotting (WB). Proteins were revealed using using antibodies against c-Myc, β-actin, or HIV-1 PR. Additional file 4:
**Figure S10.** Quantification of signal intensities in Figure 2 and 3. Bands were visualized and quantified using an Odyssey Infrared Imaging System (LI-COR Biosciences). Results are from a single experiment and are representative of at least three separate experiments. All values were normalized to β-actin levels. (A) Bar graph demonstrating levels of full-length Gag-SF in Figure 2B (B) Bar graph demonstrating levels of full-length Myc-RIPK1 in Figure 2C. (C) Bar graph demonstrating levels of full-length Myc-RIPK2 in Figure 2D. (D) Bar graph demonstrating levels of full-length RIPK1 in Figure 3A. Additional file 5:
**Figure S3.** HIV-1 PR cleaves caspase-8. HEK293T cells were transfected with a caspase-8 expression plasmid along with increasing amounts of catalytically active HIV-1 PR. Addition of SQV (5 μM) or transfection of catalytically inactive HIV PR (D25N) served as negative controls. Cells were lysed 24 hrs after transfection and total cell extracts were subjected to SDS-PAGE and immunoblotting (WB). Proteins were revealed using antibodies against caspase-8 (rabbit polyclonal), β-actin, or HIV-1 PR. Additional file 6:
**Figure S4.** Detection of cleaved RIPK1 by different antibodies. HEK293T cells were transfected with expression constructs encoding (A) Myc-RIPK1 (N-terminally tagged) or (B) RIPK1-HA (C-terminally tagged), respectively, along with increasing amounts of catalytically active HIV-1 PR. Addition of SQV (5 μM) or transfection of catalytically inactive HIV-1 PR D25N served as negative controls. Cells were lysed 24 hrs after transfection and total cell extracts were subjected to SDS-PAGE and immunoblotting (WB). Proteins were revealed using the indicated antibodies. Additional file 7:
**Figure S5.** HIV-1 PR cleaves endogenous RIPK1 in Jurkat cells. (A) Jurkat cells stable for doxycycline-(DOX)-inducible expression of firefly luciferase were treated with indicated concentrations of DOX, After 20 hours, cells were lysed by addition of one volume of Steady-Glo^R^ (Promega). Luciferase activity was measured using a standard plate luminometer (relative fluorescence units, RFU). Values represent means +/- standard deviation of triplicate cultures (means +/- SD, n= 3). (B) Jurkat cells stable for DOX-inducible expression of HIV-1 PR were treated with DOX (1 μg/ml) in the absence or presence of PR inhibitor SQV (5 μM). At indicated time points, cells were collected in lysis buffer and total cell lysates were subjected to SDS-PAGE and immunoblotting (WB). Proteins were revealed using antibodies against RIPK1, β-actin, or HIV-1 PR. (C) Jurkat cells with DOX-inducible expression of catalytically active (wild-type) or catalytically inactive HIV-1 PR (D25N) were treated with increasing concentrations of DOX. After 20 hours, cells were collected in lysis buffer and total cell lysates were analyzed by SDS-PAGE and immunoblotting (WB). Proteins were revealed using antibodies against RIPK1, β-actin, or HIV-1 PR. Arrowheads indicate cleaved RIPK1. Asterisks indicate non-specific bands. (D) Jurkat or Jurkat *RIPK1* ^-/-^ cells [58] stable for DOX-inducible expression of HIV-1 PR were treated with increasing concentrations of DOX. After 20 hours, cells were collected in lysis buffer and total cell lysates were analyzed by SDS-PAGE and immunoblotting (WB). Proteins were revealed using an antibody against RIPK1. Arrowheads indicate cleaved RIPK1. Asterisks indicate non-specific bands. Additional file 8:
**Figure S6.** Effect of discordance-associated mutations in PR on RIPK1 processing. HEK293T cells were co-transfected with an expression construct encoding My-tagged RIPK1 along with different PR mutants. Discordance-associated mutations, I54V and V82A, were tested along with unrelated mutations K20R, L63P, D30N and L90M. Transfection of wild-type PR, or the active site dead D25G mutation served as controls. Cells were either left non-treated or treated with Saquinvir (SQV, 5 μM). Cell lysates were prepared 24 hours after infection and subjected to SDS-PAGE and immunoblotting (WB). Proteins were revealed using antibodies against c-Myc, β-actin, or HIV-1 PR. Additional file 9:
**Figure S7.** RIPK1 cleavage product submitted to mass spec analysis. (A) HEK293T cells were transfected with expression plasmids encoding Myc-tagged RIPK1 along with catalytically active HIV PR (50 ng/well). Cells were lysed 24 hours after transfection and the N-terminal fragment of cleaved RIPK1 was purified from total cell extracts by immunoprecipitation with an anti-Myc antibody. Purified complexes were subjected to SDS-PAGE analysis and proteins were visualized by Coomassie staining. (B) HEK293T cells were transfected with expression plasmids encoding wild-type (wt) RIPK1 or RIPK1 with a double-mutation in the PR cleavage site (RIPK1 NQ), respectively, along with along with increasing amounts of catalytically active HIV PR. Cell lysates were subjected to SDS-PAGE and immunoblotting (WB). Proteins were revealed using antibodies against c-Myc, β-actin, or HIV-1 PR. Additional file 10:
**Figure S11.** Quantification of signal intensities in Figure 5. Bands were visualized and quantified using an Odyssey Infrared Imaging System (LI-COR Biosciences). Results are from a single experiment and are representative of at least three separate experiments. All values were normalized to β-actin levels. (A) Bar graph demonstrating levels of full-length and cleaved Myc-RIPK1 in Figure 5A. (B) Bar graph demonstrating levels of full-length and cleaved Myc-RIPK2 based in Figure 5A. (C) Bar graph demonstrating levels of full-length and cleaved Myc-RIPK1 in Figure 5C. (D) Bar graph demonstrating levels of full-length and cleaved Myc-RIPK1 in Figure 5D. (E) Bar graph demonstrating levels of full-length and cleaved Myc-RIPK1 in Figure S8. (F) Bar graph demonstrating levels of full-length and cleaved Myc-RIPK1 in Figure 5E. Additional file 11:
**Figure S8.** RIPK1 is cleaved in T cell lines during HIV-1 infection. CEM cells were infected with increasing MOIs of replication-competent HIV-1 NL4.3. Cells were either left non-treated or treated with Saquinvir (SQV, 5 μM), Raltegravir (RAL), or Nevirapine (NVP), respectively. Cell lysates were prepared 48 hours after infection and subjected to SDS-PAGE and immunoblotting (WB). Proteins were revealed using antibodies against RIKP1 (rabbit monoclonal antibody from Cell Signaling), p24, or β-actin. Additional file 12:
**Figure S9.** RIPK1 processing occurs in the absence of “incoming” PR. HEK293T cells were transiently transfected with proviral plasmids pNL4.3-Luc-Env^-^ (NIH AIDS Research Program) and psPAX2, respectively. Cells were either left non-treated or treated with Saquinvir (SQV, 5 μM). Transfection with pcDNA served as negative control. Cell lysates were prepared 20 or 40 hours after transfection and subjected to SDS-PAGE and Western blotting (WB). Proteins were revealed using antibodies against RIKP1 (rabbit antibody), or p24.

## Abbreviations

HIV-1: human immunodeficiency virus type 1
PR: HIV-1 protease
RIPK: receptor-interacting protein kinase
RT: reverse transcriptase
Gag: group-specific antigen
CA: capsid protein
KD: kinase domain
DD: death domain
ID: intermediate domain
RHIM: RIP homotypic interaction motif
CARD: caspase activation and recruitment domain
SQV: saquinavir
AZT: azidothymidine
NVP: nevirapine
RAL: raltegravir
