# Supplementary material for: HIV-1 protease cleaves the serine-threonine kinases RIPK1 and RIPK2
Source: Retrovirology. 2015 Aug 22;12:74. doi: 10.1186/s12977-015-0200-6 (PMC4546280; doi:10.1186/s12977-015-0200-6)
Supplement: Additional file 1: — Table S1. Host cell substrates of HIV-1 PR. Known host cell substrates of HIV-1 PR were curated from the scientific literature and listed according to their publication date. Supporting evidence for each substrate is specified along with respective references to original publications. [file 12977_2015_200_MOESM1_ESM.pdf]

S1 Table. Host cell substrates of HIV-1 protease.

Host cell protein	***Evidence***	***Citation***
Vimentin	In vitro, recombinant PR, microinjection	[1-4]
Desmin	In vitro, recombinant PR	[1,5]
Glia fibrillary acidic protein	In vitro, recombinant PR	[1]
Microtubule-associated protein MAP1	In vitro, recombinant PR	[6]
Microtubule-associated protein MAP2	In vitro, recombinant PR	[6]
Phosphorylase kinase	In vitro, recombinant PR	[7]
Calmodulin (Ca^{2+}-free)	In vitro, recombinant PR	[7]
Fibronectin	In vitro, recombinant PR	[8,9]
NF-κB	virus infection, in vitro (Processing of p105 precursor)	[10]
Actin	In vitro, recombinant PR, virus infection	[5,11,12]
α-actinin	In vitro, recombinant PR	[11]
Spectrin	In vitro, recombinant PR	[9,11]
Tropomyosin	In vitro, recombinant PR	[2,5,11]
Troponin C	In vitro, recombinant PR	[13]
Alzheimer amyloid precursor protein (AAP)	In vitro, recombinant PR	[13]
Pro-interleukin 1β	In vitro, recombinant PR	[13]
Myosin	In vitro, recombinant PR	[5]
Bcl-2	Virus infection, overexpression of PR by transient transfection, in vitro	[14]
Complement factor C3	In vitro, recombinant PR	[15]
eIF4G	Virus infection, overexpression of PR by transient transfection, in vitro	[16-18]
Caspase-8	Virus infection, in vitro, patient samples	[19-21]
Fimbrin	In vitro, recombinant PR, microinjection	[9]
Focal adhesion plaque kinase	In vitro, recombinant PR, microinjection	[9]
Talin	In vitro, recombinant PR, microinjection	[9]
Filamin	In vitro, recombinant PR, microinjection	[9]
Integrin α3	In vitro, recombinant PR, microinjection	[9]
Integrin β4	In vitro, recombinant PR, microinjection	[9]
NDR1	Virus infection, overexpression of PR by transient transfection, in vitro, viron-associated host cell kinase	[22]
NDR2	Virus infection, overexpression of	[22]

	PR by transient transfection, in vitro, viron-associated host cell kinase	
PABP [poly(A)-binding protein]	Virus infection, overexpression of PR by transient transfection, in vitro	[17,23]
Lyric	Virus infection, Gag-interacting protein, viron-associated host cell protein	[24]
GCN2	Virus infection, overexpression of PR by transient transfection, in vitro	[25]
eIF3D	overexpression of PR by transient transfection, siRNA, in vitro	[26]
DDX5	In vitro, recombinant PR	[27]

1. Shoeman RL, Honer B, Stoller TJ, Kesselmeier C, Miedel MC, et al. (1990) Human immunodeficiency virus type 1 protease cleaves the intermediate filament proteins vimentin, desmin, and glial fibrillary acidic protein. Proc Natl Acad Sci U S A 87: 6336-6340.
2. Honer B, Shoeman RL, Traub P (1992) Degradation of cytoskeletal proteins by the human immunodeficiency virus type 1 protease. Cell Biol Int Rep 16: 603-612.
3. Shoeman RL, Huttermann C, Hartig R, Traub P (2001) Amino-terminal polypeptides of vimentin are responsible for the changes in nuclear architecture associated with human immunodeficiency virus type 1 protease activity in tissue culture cells. Mol Biol Cell 12: 143-154.
4. Shoeman RL, Mothes E, Kesselmeier C, Traub P (1990) Intermediate filament assembly and stability in vitro: effect and implications of the removal of head and tail domains of vimentin by the human immunodeficiency virus type 1 protease. Cell Biol Int Rep 14: 583-594.
5. Shoeman RL, Sachse C, Honer B, Mothes E, Kaufmann M, et al. (1993) Cleavage of human and mouse cytoskeletal and sarcomeric proteins by human immunodeficiency virus type 1 protease. Actin, desmin, myosin, and tropomyosin. Am J Pathol 142: 221-230.
6. Wallin M, Deinum J, Goobar L, Danielson UH (1990) Proteolytic cleavage of microtubule-associated proteins by retroviral proteinases. J Gen Virol 71 (Pt 9): 1985-1991.
7. Daube H, Billich A, Mann K, Schramm HJ (1991) Cleavage of phosphorylase kinase and calcium-free calmodulin by HIV-1 protease. Biochem Biophys Res Commun 178: 892-898.
8. Oswald M, von der Helm K (1991) Fibronectin is a non-viral substrate for the HIV proteinase. FEBS Lett 292: 298-300.
9. Shoeman RL, Hartig R, Hauses C, Traub P (2002) Organization of focal adhesion plaques is disrupted by action of the HIV-1 protease. Cell Biol Int 26: 529-539.
10. Riviere Y, Blank V, Kourilsky P, Israel A (1991) Processing of the precursor of NF-kappa B by the HIV-1 protease during acute infection. Nature 350: 625-626.
11. Shoeman RL, Kesselmier C, Mothes E, Honer B, Traub P (1991) Non-viral cellular substrates for human immunodeficiency virus type 1 protease. FEBS Lett 278: 199-203.
12. Adams LD, Tomasselli AG, Robbins P, Moss B, Heinrikson RL (1992) HIV-1 protease cleaves actin during acute infection of human T-lymphocytes. AIDS Res Hum Retroviruses 8: 291-295.
13. Tomasselli AG, Hui JO, Adams L, Chosay J, Lowery D, et al. (1991) Actin, troponin C, Alzheimer amyloid precursor protein and pro-interleukin 1 beta as substrates

of the protease from human immunodeficiency virus. J Biol Chem 266: 14548-14553.
14. Strack PR, Frey MW, Rizzo CJ, Cordova B, George HJ, et al. (1996) Apoptosis mediated by HIV protease is preceded by cleavage of Bcl-2. Proc Natl Acad Sci U S A 93: 9571-9576.
15. Kisselev AF, Mentele R, von der Helm K (1997) Cleavage of the complement system C3 component by HIV-1 proteinase. Biol Chem 378: 439-442.
16. Ventoso I, Blanco R, Perales C, Carrasco L (2001) HIV-1 protease cleaves eukaryotic initiation factor 4G and inhibits cap-dependent translation. Proc Natl Acad Sci U S A 98: 12966-12971.
17. Castello A, Franco D, Moral-Lopez P, Berlanga JJ, Alvarez E, et al. (2009) HIV- 1 protease inhibits Cap- and poly(A)-dependent translation upon eIF4GI and PABP cleavage. PLoS One 4: e7997.
18. Ohlmann T, Prevot D, Decimo D, Roux F, Garin J, et al. (2002) In vitro cleavage of eIF4GI but not eIF4GII by HIV-1 protease and its effects on translation in the rabbit reticulocyte lysate system. J Mol Biol 318: 9-20.
19. Nie Z, Phenix BN, Lum JJ, Alam A, Lynch DH, et al. (2002) HIV-1 protease processes procaspase 8 to cause mitochondrial release of cytochrome c, caspase cleavage and nuclear fragmentation. Cell Death Differ 9: 1172-1184.
20. Nie Z, Bren GD, Vlahakis SR, Schimnich AA, Brenchley JM, et al. (2007) Human immunodeficiency virus type 1 protease cleaves procaspase 8 in vivo. J Virol 81: 6947-6956.
21. Nie Z, Bren GD, Rizza SA, Badley AD (2008) HIV Protease Cleavage of Procaspase 8 is Necessary for Death of HIV-Infected Cells. Open Virol J 2: 1-7.
22. Devroe E, Silver PA, Engelman A (2005) HIV-1 incorporates and proteolytically processes human NDR1 and NDR2 serine-threonine kinases. Virology 331: 181-189.
23. Alvarez E, Castello A, Menendez-Arias L, Carrasco L (2006) HIV protease cleaves poly(A)-binding protein. Biochem J 396: 219-226.
24. Engeland CE, Oberwinkler H, Schumann M, Krause E, Muller GA, et al. (2011) The cellular protein lyric interacts with HIV-1 Gag. J Virol 85: 13322-13332.
25. del Pino J, Jimenez JL, Ventoso I, Castello A, Munoz-Fernandez MA, et al. (2012) GCN2 has inhibitory effect on human immunodeficiency virus-1 protein synthesis and is cleaved upon viral infection. PLoS One 7: e47272.
26. Jager S, Cimermancic P, Gulbahce N, Johnson JR, McGovern KE, et al. (2012) Global landscape of HIV-human protein complexes. Nature 481: 365-370.
27. Impens F, Timmerman E, Staes A, Moens K, Arien KK, et al. (2012) A catalogue of putative HIV-1 protease host cell substrates. Biol Chem 393: 915-931.
