# Supplementary figures and images for: HIV-1 protease cleaves the serine-threonine kinases RIPK1 and RIPK2
Source: Retrovirology. 2015 Aug 22;12:74. doi: 10.1186/s12977-015-0200-6 (PMC4546280; doi:10.1186/s12977-015-0200-6)

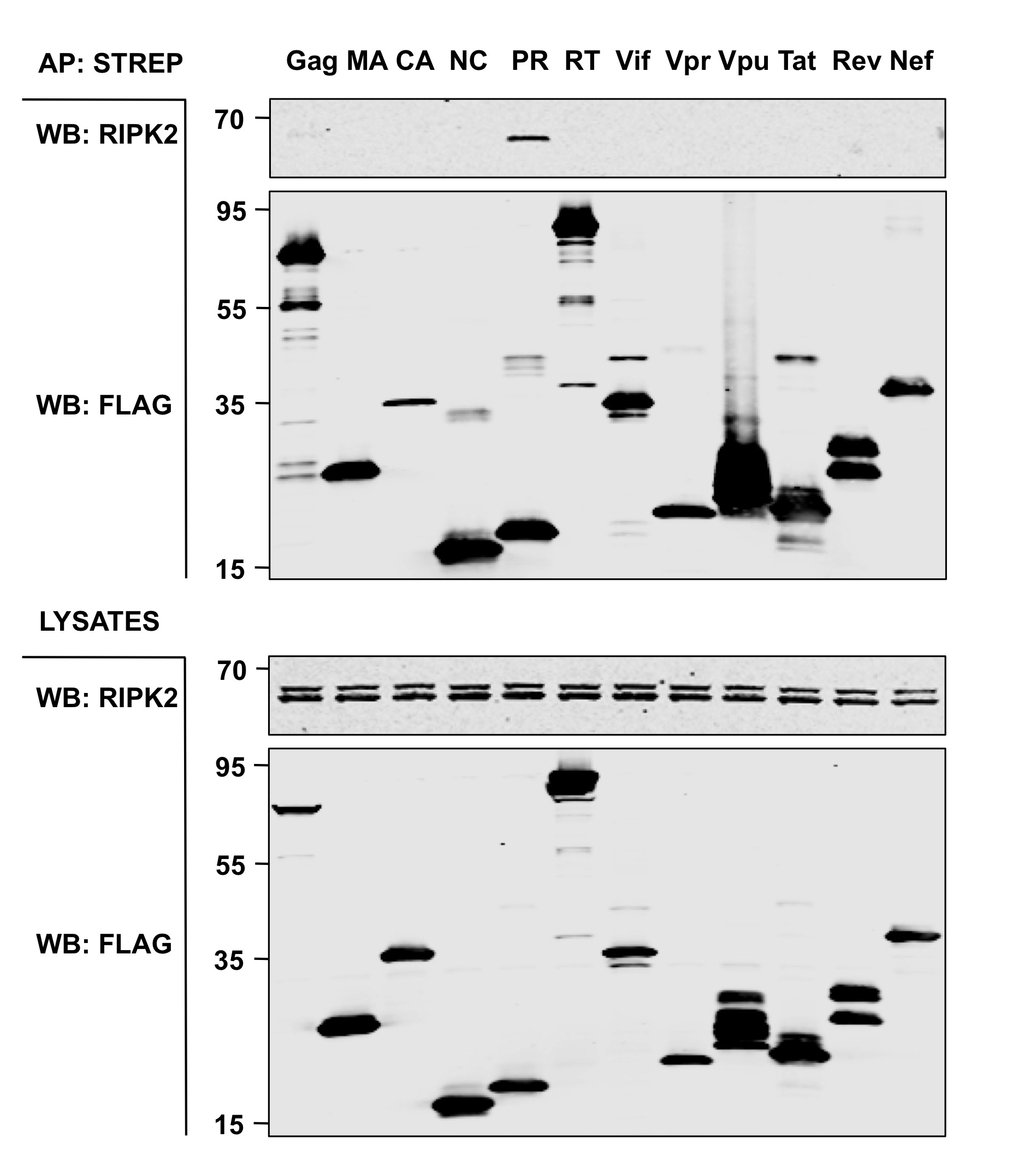

Supplement: Additional file 2: — Figure S1. Endogenous RIPK2 specifically binds HIV-1 PR. Various SF-tagged HIV-proteins were expressed by transfection in HEK293T cells. After 24 hours, proteins were affinity purified from cleared cell lysates with Dynabeads Streptavidin. Affinity purifications (APs) or total lysates were subjected to SDS-PAGE and Western blotting (WB). Proteins were revealed using antibodies against RIPK2 (BD Transduction Laboratories), or FLAG (Sigma). [file 12977_2015_200_MOESM2_ESM.tiff]

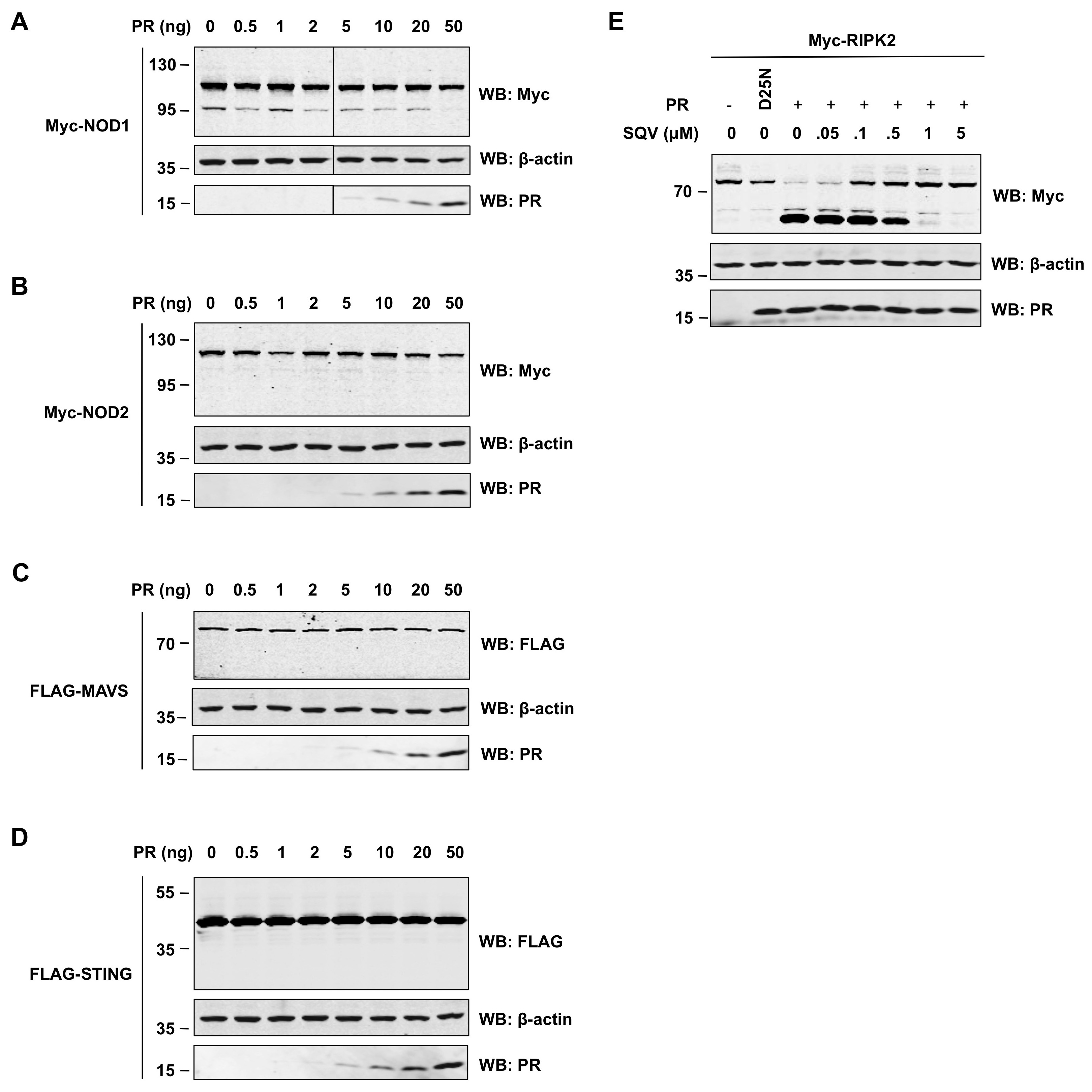

Supplement: Additional file 3: — Figure S2. HIV-1 PR does not cleave unrelated proteins. (A)-(D) HEK293T cells were transfected with plasmids encoding Myc-tagged NOD1 (A), and NOD2 (B), or FLAG-tagged MAVS (C), and STING (D) along with increasing amounts of plasmid encoding HIV-1 PR. After 24 hours, cells were collected in lysis buffer and samples were subjected to SDS-PAGE and WB analysis. Proteins were revealed using antibodies against c-Myc, FLAG, β-actin, or HIV-1 PR. (E) Dose-dependent inhibition of RIPK2 cleavage. HEK293T cells were transfected with Myc-RIPK2 in the absence (-) or presence (+) of catalytically active HIV PR (PR) or catalytically inactive HIV PR (D25N) (10 ng/well) and increasing concentrations of PR inhibitor SQV. Cell lysates were subjected to SDS-PAGE and immunoblotting (WB). Proteins were revealed using using antibodies against c-Myc, β-actin, or HIV-1 PR. [file 12977_2015_200_MOESM3_ESM.tiff]

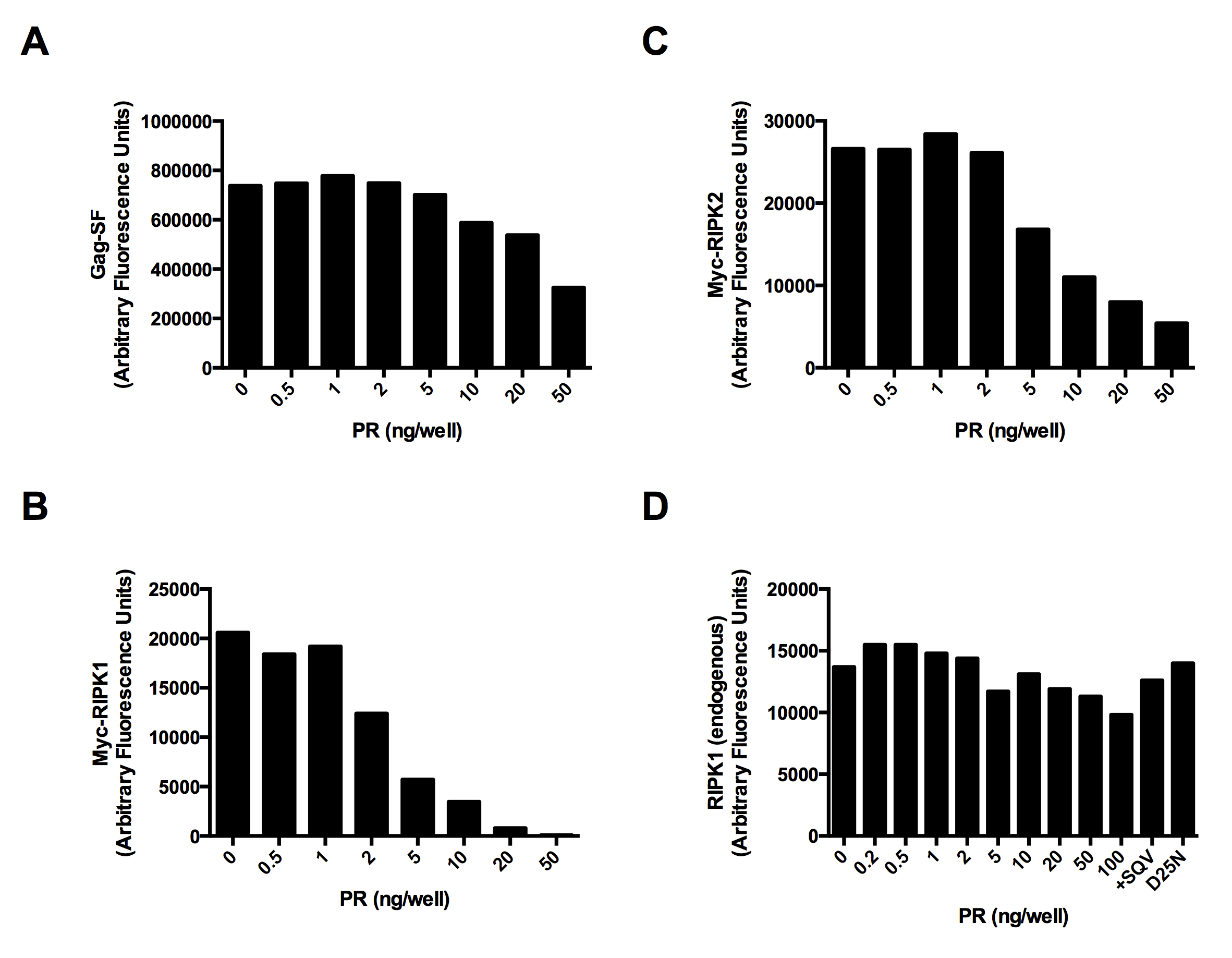

Supplement: Additional file 4: — Figure S10. Quantification of signal intensities in Figure 2 and 3. Bands were visualized and quantified using an Odyssey Infrared Imaging System (LI-COR Biosciences). Results are from a single experiment and are representative of at least three separate experiments. All values were normalized to β-actin levels. (A) Bar graph demonstrating levels of full-length Gag-SF in Figure 2B (B) Bar graph demonstrating levels of full-length Myc-RIPK1 in Figure 2C. (C) Bar graph demonstrating levels of full-length Myc-RIPK2 in Figure 2D. (D) Bar graph demonstrating levels of full-length RIPK1 in Figure 3A. [file 12977_2015_200_MOESM4_ESM.tiff]

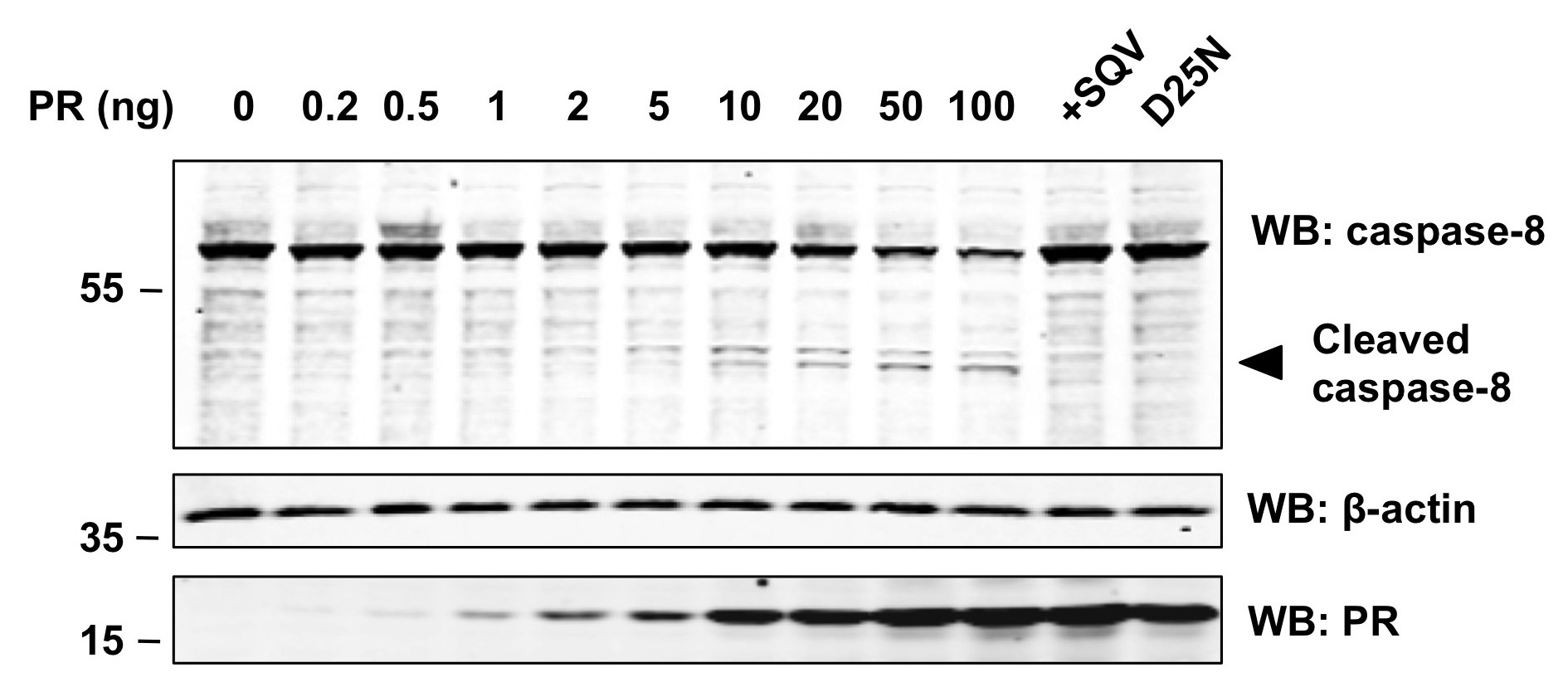

Supplement: Additional file 5: — Figure S3. HIV-1 PR cleaves caspase-8. HEK293T cells were transfected with a caspase-8 expression plasmid along with increasing amounts of catalytically active HIV-1 PR. Addition of SQV (5 μM) or transfection of catalytically inactive HIV PR (D25N) served as negative controls. Cells were lysed 24 hrs after transfection and total cell extracts were subjected to SDS-PAGE and immunoblotting (WB). Proteins were revealed using antibodies against caspase-8 (rabbit polyclonal), β-actin, or HIV-1 PR. [file 12977_2015_200_MOESM5_ESM.tiff]

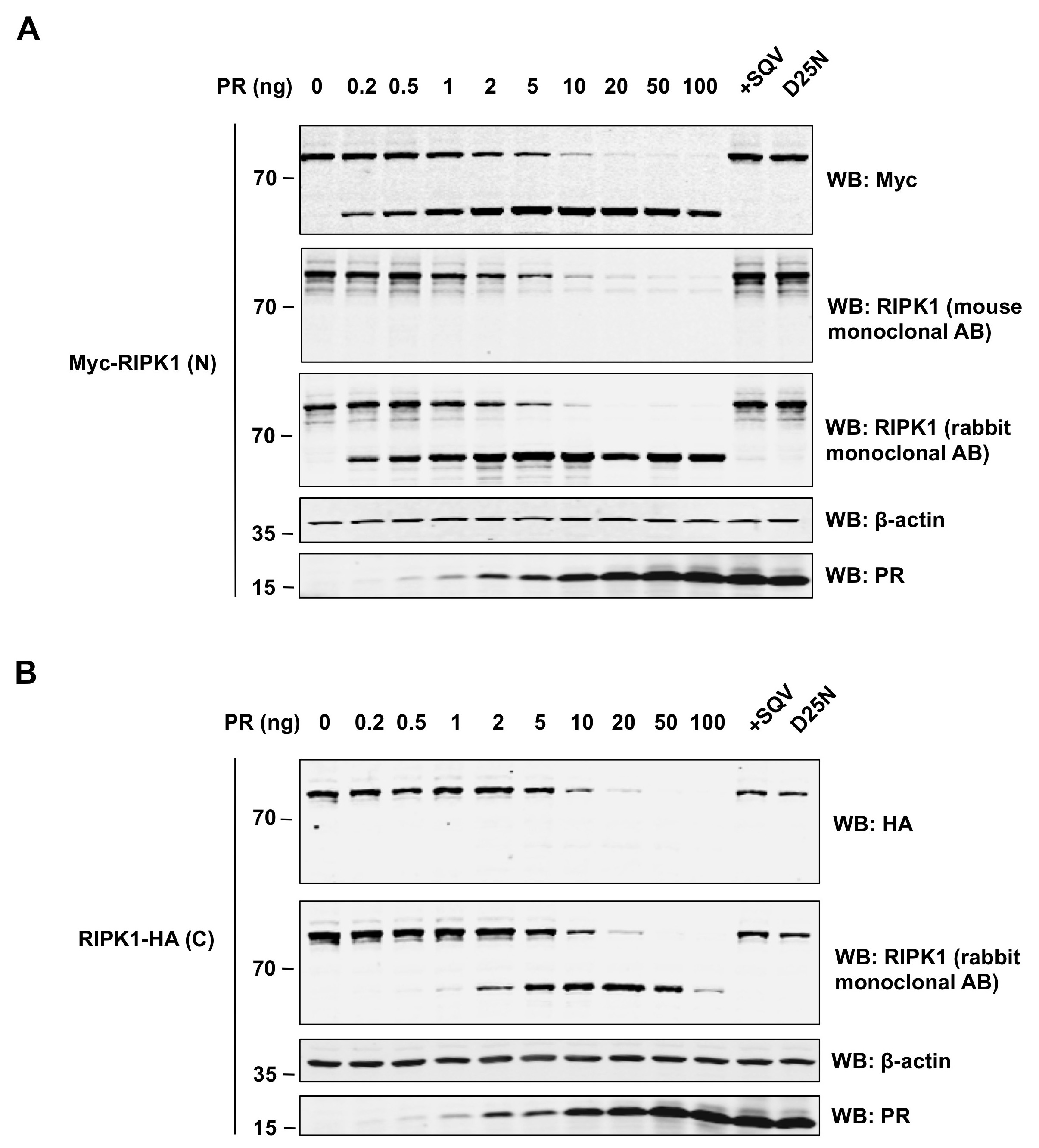

Supplement: Additional file 6: — Figure S4. Detection of cleaved RIPK1 by different antibodies. HEK293T cells were transfected with expression constructs encoding (A) Myc-RIPK1 (N-terminally tagged) or (B) RIPK1-HA (C-terminally tagged), respectively, along with increasing amounts of catalytically active HIV-1 PR. Addition of SQV (5 μM) or transfection of catalytically inactive HIV-1 PR D25N served as negative controls. Cells were lysed 24 hrs after transfection and total cell extracts were subjected to SDS-PAGE and immunoblotting (WB). Proteins were revealed using the indicated antibodies. [file 12977_2015_200_MOESM6_ESM.tiff]

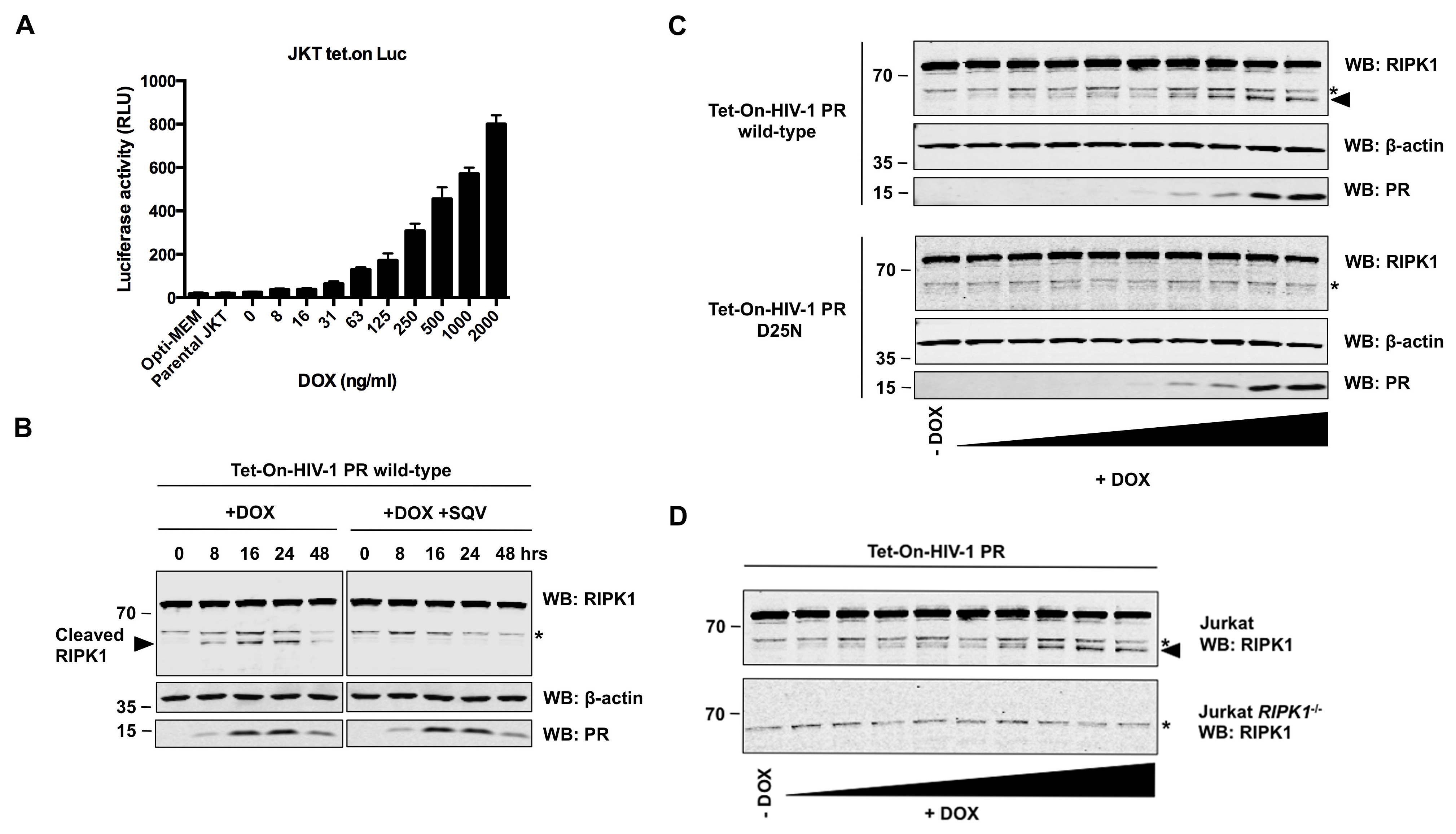

Supplement: Additional file 7: — Figure S5. HIV-1 PR cleaves endogenous RIPK1 in Jurkat cells. (A) Jurkat cells stable for doxycycline-(DOX)-inducible expression of firefly luciferase were treated with indicated concentrations of DOX, After 20 hours, cells were lysed by addition of one volume of Steady-GloR (Promega). Luciferase activity was measured using a standard plate luminometer (relative fluorescence units, RFU). Values represent means +/- standard deviation of triplicate cultures (means +/- SD, n= 3). (B) Jurkat cells stable for DOX-inducible expression of HIV-1 PR were treated with DOX (1 μg/ml) in the absence or presence of PR inhibitor SQV (5 μM). At indicated time points, cells were collected in lysis buffer and total cell lysates were subjected to SDS-PAGE and immunoblotting (WB). Proteins were revealed using antibodies against RIPK1, β-actin, or HIV-1 PR. (C) Jurkat cells with DOX-inducible expression of catalytically active (wild-type) or catalytically inactive HIV-1 PR (D25N) were treated with increasing concentrations of DOX. After 20 hours, cells were collected in lysis buffer and total cell lysates were analyzed by SDS-PAGE and immunoblotting (WB). Proteins were revealed using antibodies against RIPK1, β-actin, or HIV-1 PR. Arrowheads indicate cleaved RIPK1. Asterisks indicate non-specific bands. (D) Jurkat or Jurkat RIPK1 -/- cells [58] stable for DOX-inducible expression of HIV-1 PR were treated with increasing concentrations of DOX. After 20 hours, cells were collected in lysis buffer and total cell lysates were analyzed by SDS-PAGE and immunoblotting (WB). Proteins were revealed using an antibody against RIPK1. Arrowheads indicate cleaved RIPK1. Asterisks indicate non-specific bands. [file 12977_2015_200_MOESM7_ESM.tiff]

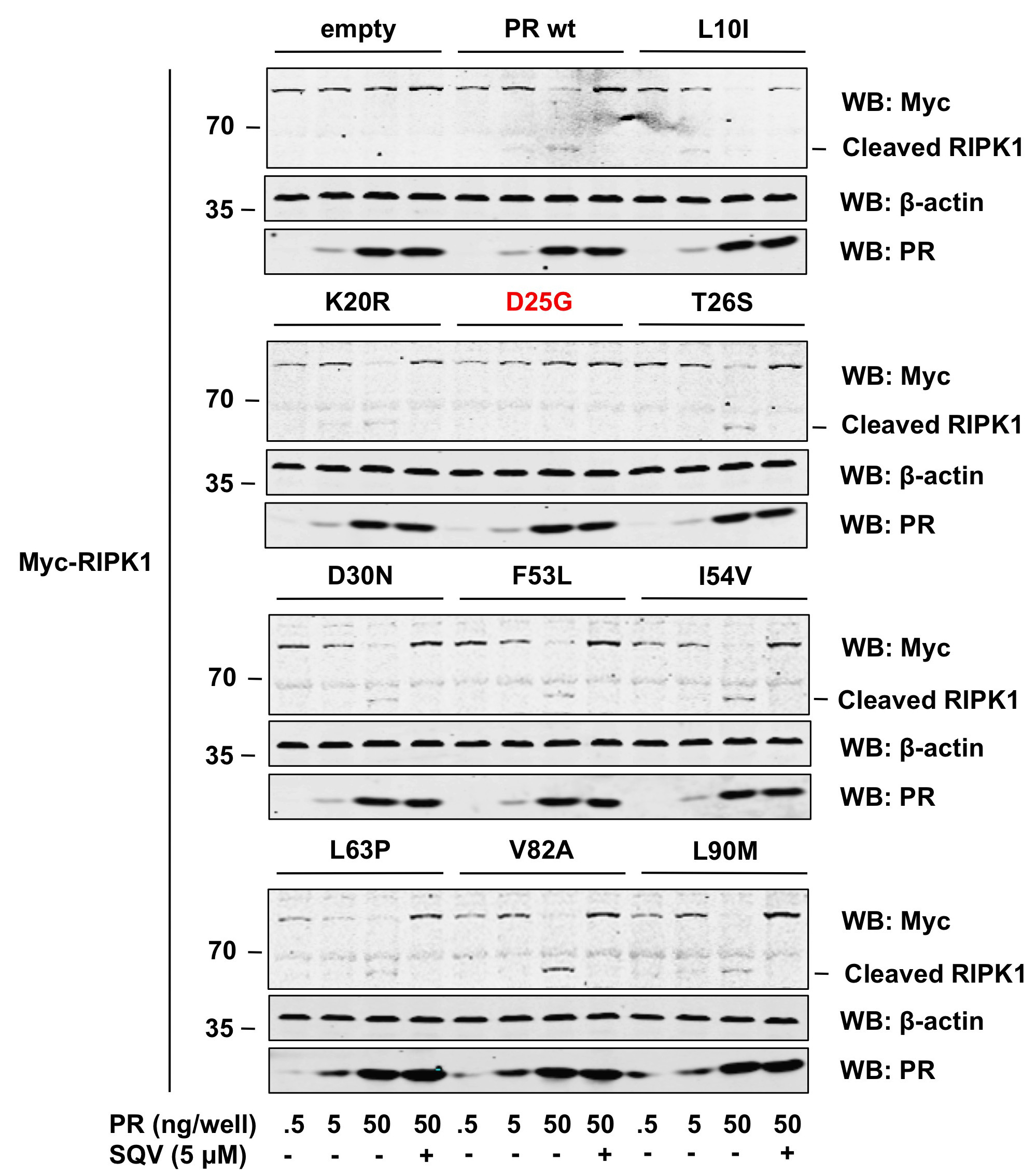

Supplement: Additional file 8: — Figure S6. Effect of discordance-associated mutations in PR on RIPK1 processing. HEK293T cells were co-transfected with an expression construct encoding My-tagged RIPK1 along with different PR mutants. Discordance-associated mutations, I54V and V82A, were tested along with unrelated mutations K20R, L63P, D30N and L90M. Transfection of wild-type PR, or the active site dead D25G mutation served as controls. Cells were either left non-treated or treated with Saquinvir (SQV, 5 μM). Cell lysates were prepared 24 hours after infection and subjected to SDS-PAGE and immunoblotting (WB). Proteins were revealed using antibodies against c-Myc, β-actin, or HIV-1 PR. [file 12977_2015_200_MOESM8_ESM.tiff]

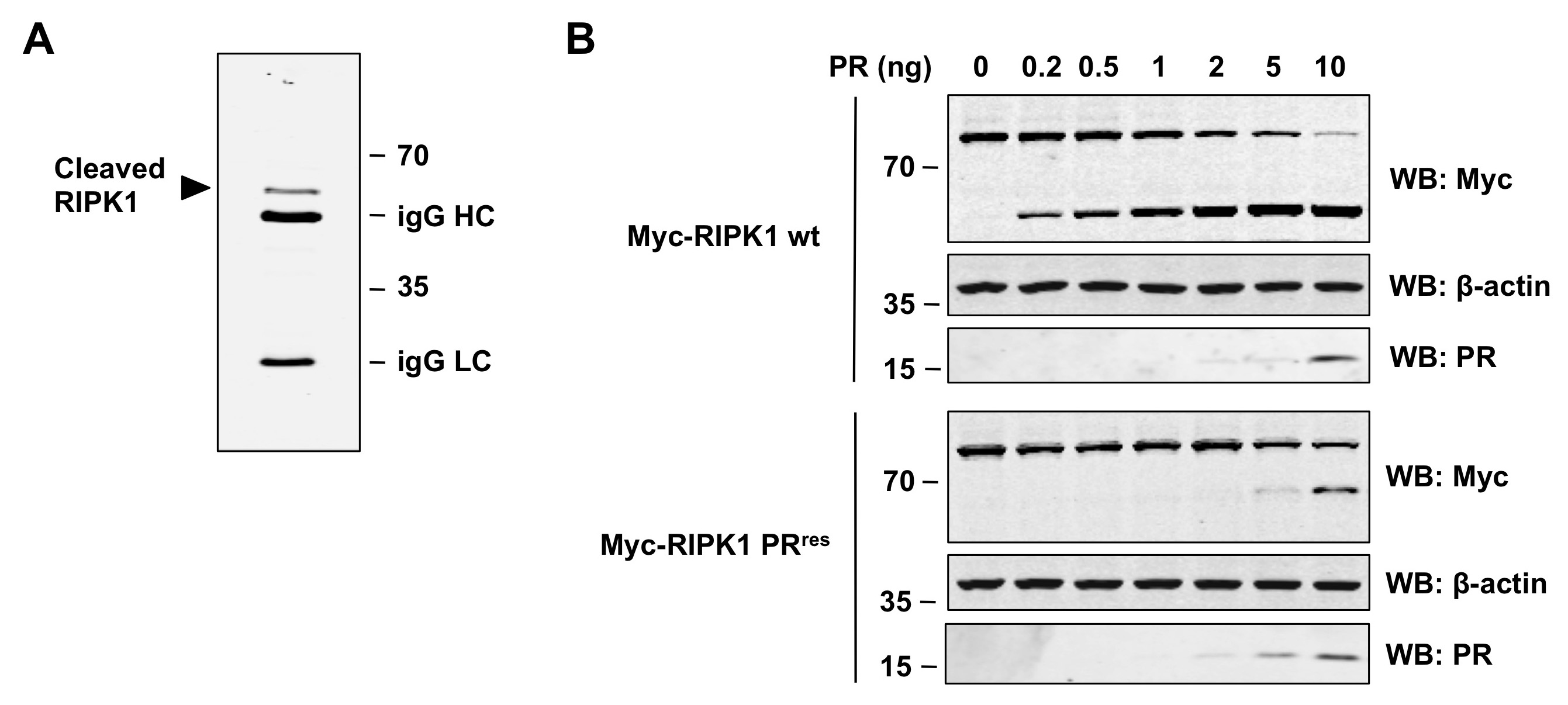

Supplement: Additional file 9: — Figure S7. RIPK1 cleavage product submitted to mass spec analysis. (A) HEK293T cells were transfected with expression plasmids encoding Myc-tagged RIPK1 along with catalytically active HIV PR (50 ng/well). Cells were lysed 24 hours after transfection and the N-terminal fragment of cleaved RIPK1 was purified from total cell extracts by immunoprecipitation with an anti-Myc antibody. Purified complexes were subjected to SDS-PAGE analysis and proteins were visualized by Coomassie staining. (B) HEK293T cells were transfected with expression plasmids encoding wild-type (wt) RIPK1 or RIPK1 with a double-mutation in the PR cleavage site (RIPK1 NQ), respectively, along with along with increasing amounts of catalytically active HIV PR. Cell lysates were subjected to SDS-PAGE and immunoblotting (WB). Proteins were revealed using antibodies against c-Myc, β-actin, or HIV-1 PR. [file 12977_2015_200_MOESM9_ESM.tiff]

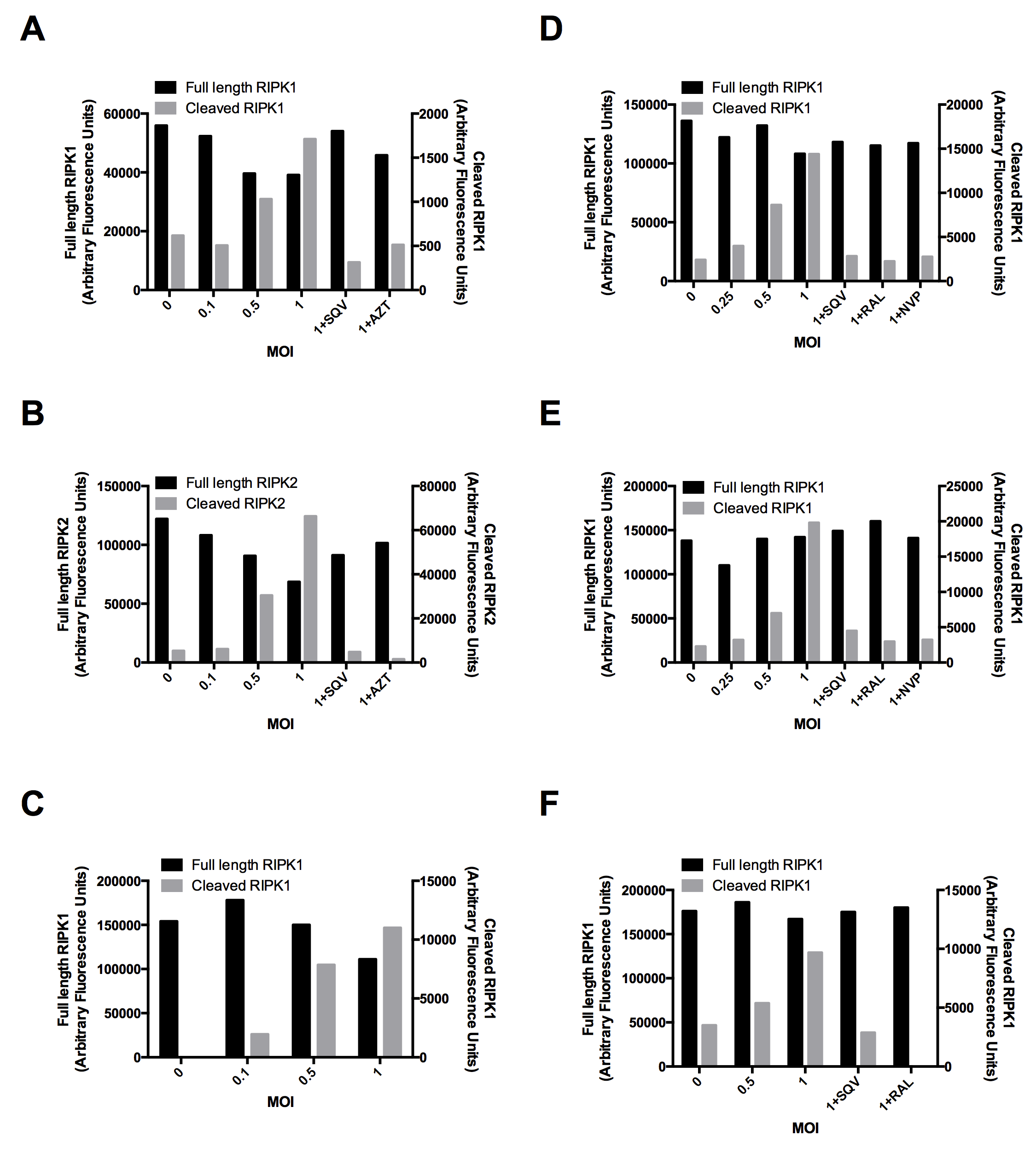

Supplement: Additional file 10: — Figure S11. Quantification of signal intensities in Figure 5. Bands were visualized and quantified using an Odyssey Infrared Imaging System (LI-COR Biosciences). Results are from a single experiment and are representative of at least three separate experiments. All values were normalized to β-actin levels. (A) Bar graph demonstrating levels of full-length and cleaved Myc-RIPK1 in Figure 5A. (B) Bar graph demonstrating levels of full-length and cleaved Myc-RIPK2 based in Figure 5A. (C) Bar graph demonstrating levels of full-length and cleaved Myc-RIPK1 in Figure 5C. (D) Bar graph demonstrating levels of full-length and cleaved Myc-RIPK1 in Figure 5D. (E) Bar graph demonstrating levels of full-length and cleaved Myc-RIPK1 in Figure S8. (F) Bar graph demonstrating levels of full-length and cleaved Myc-RIPK1 in Figure 5E. [file 12977_2015_200_MOESM10_ESM.tiff]

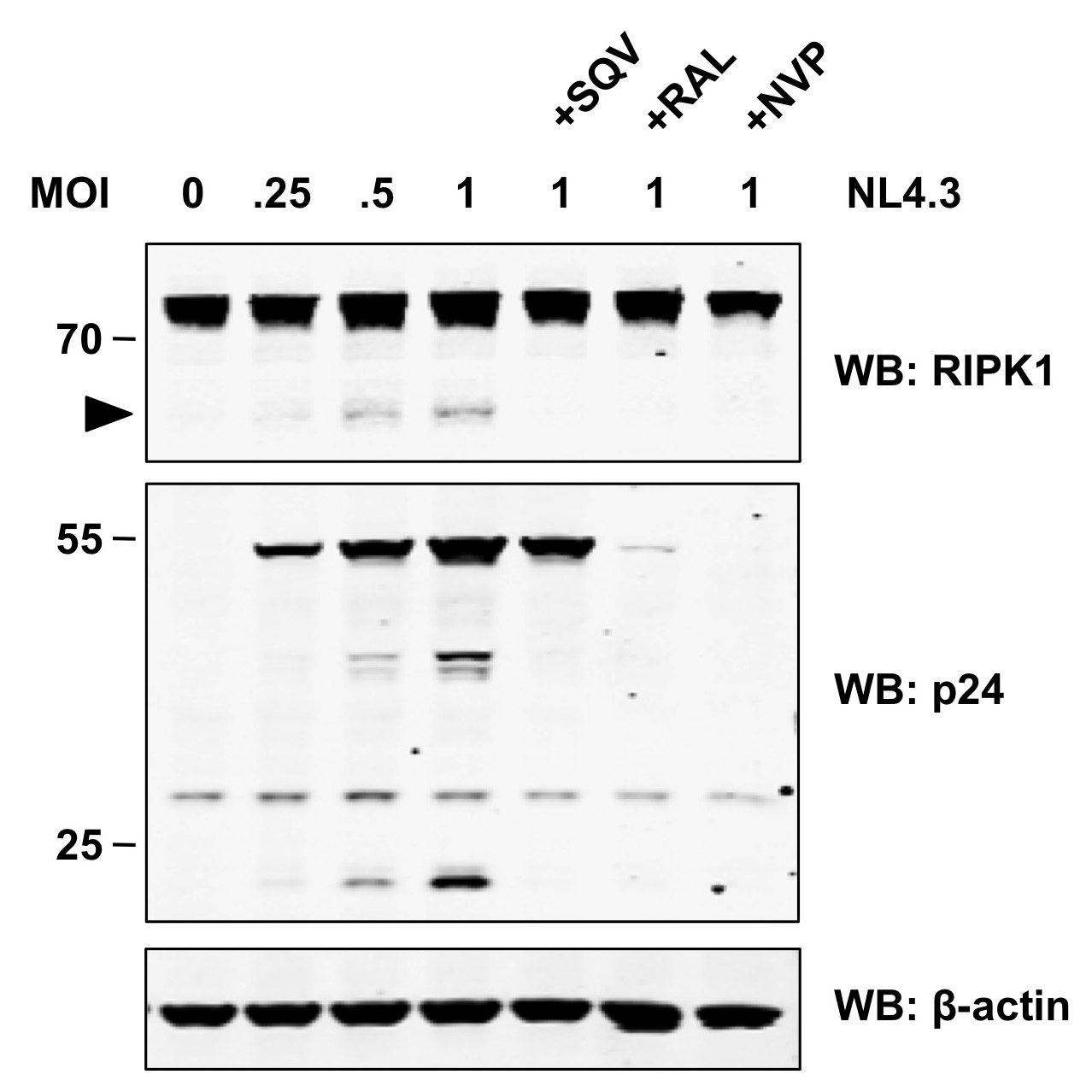

Supplement: Additional file 11: — Figure S8. RIPK1 is cleaved in T cell lines during HIV-1 infection. CEM cells were infected with increasing MOIs of replication-competent HIV-1 NL4.3. Cells were either left non-treated or treated with Saquinvir (SQV, 5 μM), Raltegravir (RAL), or Nevirapine (NVP), respectively. Cell lysates were prepared 48 hours after infection and subjected to SDS-PAGE and immunoblotting (WB). Proteins were revealed using antibodies against RIKP1 (rabbit monoclonal antibody from Cell Signaling), p24, or β-actin. [file 12977_2015_200_MOESM11_ESM.tiff]

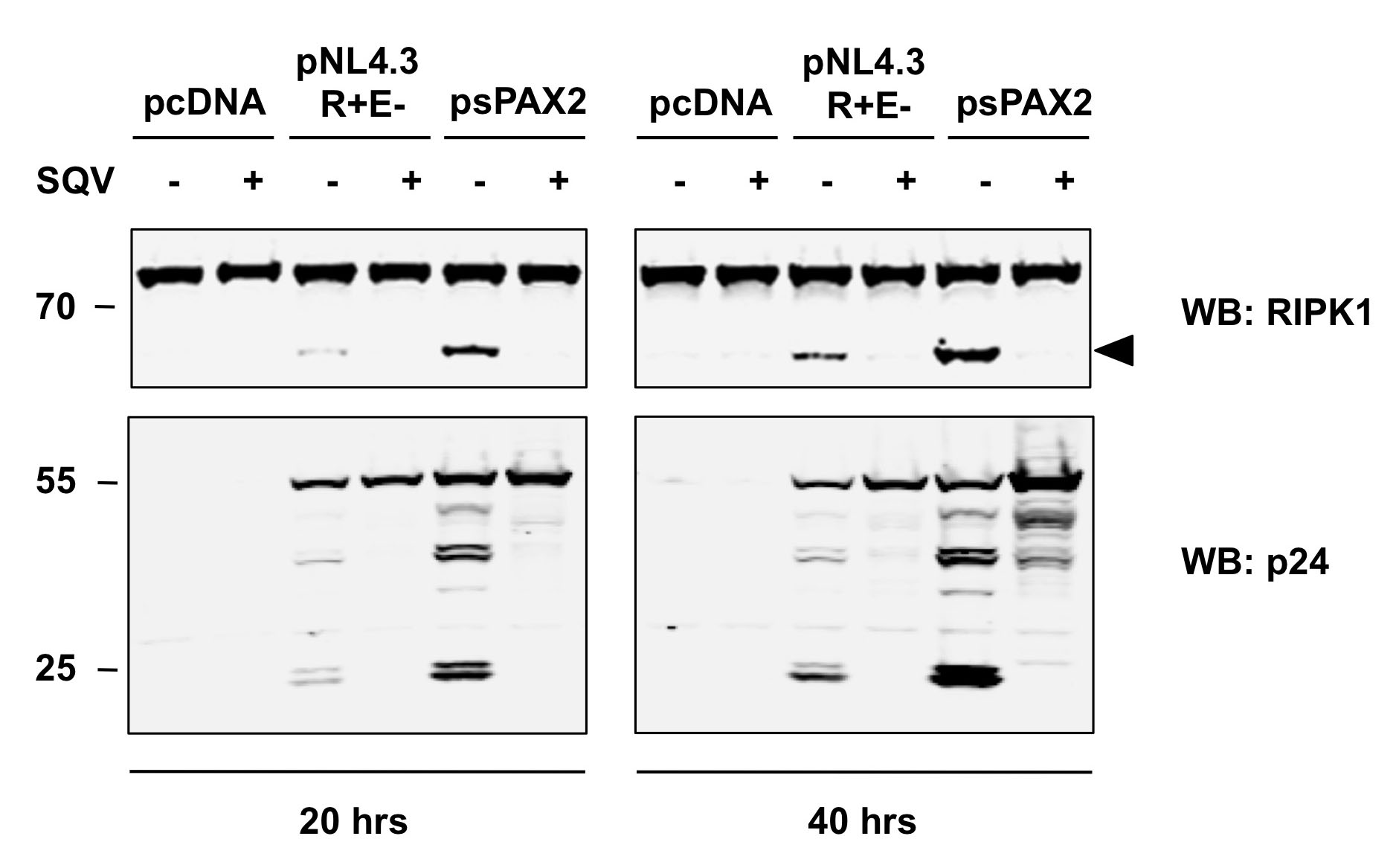

Supplement: Additional file 12: — Figure S9. RIPK1 processing occurs in the absence of “incoming” PR. HEK293T cells were transiently transfected with proviral plasmids pNL4.3-Luc-Env- (NIH AIDS Research Program) and psPAX2, respectively. Cells were either left non-treated or treated with Saquinvir (SQV, 5 μM). Transfection with pcDNA served as negative control. Cell lysates were prepared 20 or 40 hours after transfection and subjected to SDS-PAGE and Western blotting (WB). Proteins were revealed using antibodies against RIKP1 (rabbit antibody), or p24. [file 12977_2015_200_MOESM12_ESM.tiff]
